# Mitochondrial metabolic rewiring sensitizes mTORC1 inhibitor persister cells to cuproptosis

**DOI:** 10.1172/jci.insight.187448

**Published:** 2025-11-24

**Authors:** Heng Du, Heng-Jia Liu, Magdalena Losko, Yu Chi Yang, Min Yuan, Elizabeth P. Henske, John M. Asara, Mallika Singh, David J. Kwiatkowski

**Affiliations:** 1 Department of Thoracic Surgery and; 2 Lung Cancer Center, West China Hospital, Sichuan University, Chengdu, China.; 3 Division of Pulmonary and Critical Care Medicine, Brigham and Women’s Hospital, Boston, Massachusetts, USA.; 4 Centre for Infection Immunity and Cancer, Zhejiang University-University of Edinburgh Institute, Zhejiang University School of Medicine, Zhejiang University, Haining, Zhejiang, China.; 5 Edinburgh Medical School: Biomedical Sciences, College of Medicine and Veterinary Medicine, University of Edinburgh, Edinburgh, United Kingdom.; 6 Department of Biology, Revolution Medicines Inc., Redwood City, California, USA.; 7 Division of Signal Transduction, Beth Israel Deaconess Medical Center, Boston, Massachusetts, USA.; 8 Department of Medicine, Harvard Medical School, Boston, Massachusetts, USA.

**Keywords:** Metabolism, Oncology, Cancer, Epigenetics, Mitochondria

## Abstract

Therapeutics blocking PI3K/mTOR complex 1 (mTORC1) are commonly used for tumor treatment, and at times achieve major responses, yet minimal residual disease (MRD) persists, leading to tumor relapse. We developed multiple MRD models both in vitro (rapamycin persistent, RP) and in vivo after mTORC1 inhibition. All 11 RP/MRD cell lines showed complete growth and signaling insensitivity to rapamycin but variable sensitivity to bi-steric mTORC1 inhibitors, with *Mtor^S2035^* mutations identified in 4 of 7 RP cell lines. Multiomic analyses identified a pronounced shift toward oxidative phosphorylation and away from glycolysis with increased mitochondrial number in all RP/MRD models. MYC and SWI/SNF expression was significantly enhanced. Both the SWI/SNF inhibitor AU-15330 and the mitochondrial complex I oxidative phosphorylation inhibitor IACS-010759 showed pronounced synergy with bi-steric mTORC1 inhibitors to cause cuproptotic cell death in RP/MRD cells, suggesting these combinations as a potential patient treatment strategy for rapalog resistance.

## Abstract 2

mTORC1 inhibitor persistent cells rely on mitochondria and are sensitive to copper induced cell death.

## Graphical abstract

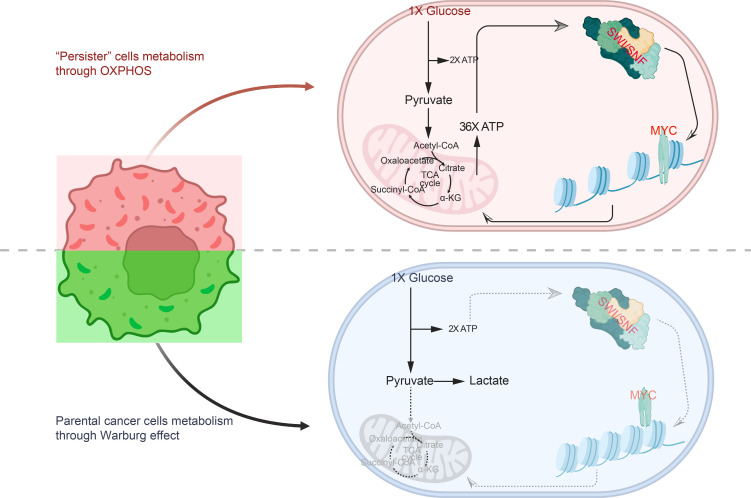

## Introduction

The PI3K/AKT/mTOR pathway is a vital intracellular signaling cascade, orchestrating various cellular processes, including growth, proliferation, metabolism, and survival (1, 2). Dysregulation of this pathway occurs frequently in the pathogenesis of cancer and other diseases (3). The tuberous sclerosis complex (TSC) protein complex, consisting of TSC1 (hamartin), TSC2 (tuberin), and TBC1D7, functions as a key negative regulator of mTOR complex 1 (mTORC1) signaling by converting the small GTPase Rheb into its inactive GDP-bound form (4). In turn, mTORC1 regulates numerous downstream processes, including protein synthesis, nucleotide and lipid metabolism, macromolecular assembly (including ribosomes), and autophagy, in response to diverse environmental cues, such as nutrient availability and growth factors (5–9). Chronic activation of mTORC1, secondary to inactivating mutations in TSC1 or TSC2, occurs in the syndromic tumors of TSC, as well as various malignancies, including bladder cancer (BLCA), hepatocellular cancer (HCC), renal cell carcinoma (RCC), and malignant perivascular epithelioid cell tumor (PEComa) (10–12).

Several generations of mTORC1 inhibitors have been developed: rapamycin and derivatives (rapalogs), kinase inhibitors including sapanisertib (13), and bi-steric mTORC1-selective inhibitors (14). Although rapalog therapy causes tumor reduction in lymphangioleiomyomatosis (LAM) and angiomyolipoma (AML), each due predominantly to inactivating TSC2 mutations, there was a dramatic tumor regrowth after treatment cessation (15). Treatment of multiple TSC mouse models with rapalogs also reduces tumor growth to a major extent (>90%), but the tumors recur when treatment is discontinued (13, 16–18). Hence these observations in both mouse models and from clinical trials indicate that “persister cells” remain after rapalog therapy and will regrow when treatment ends.

Minimal residual disease (MRD) refers to the small number of cancer cells that remain in the body after targeted treatment, which are often hard to detect by standard clinical methods and provide a source of cells that can evolve into fully resistant cancer cells that progress despite ongoing treatment (19). Multiple mechanisms contribute to development of MRD, including genetic, and nongenetic, including expression and epigenetic alterations (20–24). The SWI/SNF chromatin remodeling complex, with multiple protein subunits, including BRG1/BRM-associated factor (BAF), polybromo-associated BAF, and noncanonical BAF, regulates gene expression by modulating chromatin structure and accessibility in an ATP-dependent manner (25). Dysregulation of SWI/SNF complex components is commonly observed in various cancers, leads to aberrant gene expression patterns, and has been implicated in driving drug resistance (26, 27).

Mitochondria play a multifaceted role in tumor cells, impacting various aspects of cancer biology, most importantly energy metabolism (28). Alterations in mitochondrial function and dynamics are frequently observed in cancer cells, most commonly metabolic reprogramming with increased aerobic glycolysis and reduced oxidative phosphorylation (OXPHOS) (29). In addition, mitochondria contribute to drug resistance mechanisms in cancer cells in several ways, including modulation of apoptosis, alteration of reactive oxygen species (ROS) levels, and regulation of drug efflux pumps (30, 31).

In this study, we used chronic treatment with rapamycin on TSC1/TSC2-mutant tumor cell lines, including human BLCA, HCC, and AML, and mouse RCC, to generate rapamycin-persistent (RP) derivatives. Two MRD cell lines derived from a syngeneic mouse model treated with rapamycin or the bi-steric mTORC1 inhibitor RMC-6272 were also generated (17). *Mtor* mutations were identified in 4 of the 7 RP models. We employed a multiomics approach to investigate how long-term mTORC1 suppression rewired the metabolism of these RP/MRD lines. We found that there was enhancement of SWI/SNF activity, a marked increase in MYC expression, and a pronounced shift from glycolysis to aerobic OXPHOS. mTORC1 bi-steric treatment of the RP/MRD cell lines combined with either an SWI/SNF inhibitor or an inhibitor of OXPHOS showed a strong synergistic effect, inducing cuproptosis and cell death. These observations suggest that these combination treatments could be effective for treatment or suppression of rapamycin persistence and MRD development following long-term rapamycin treatment in patients.

## Results

### Chronic mTORC1 suppression generates a persistent phenotype.

**Figure 1:**
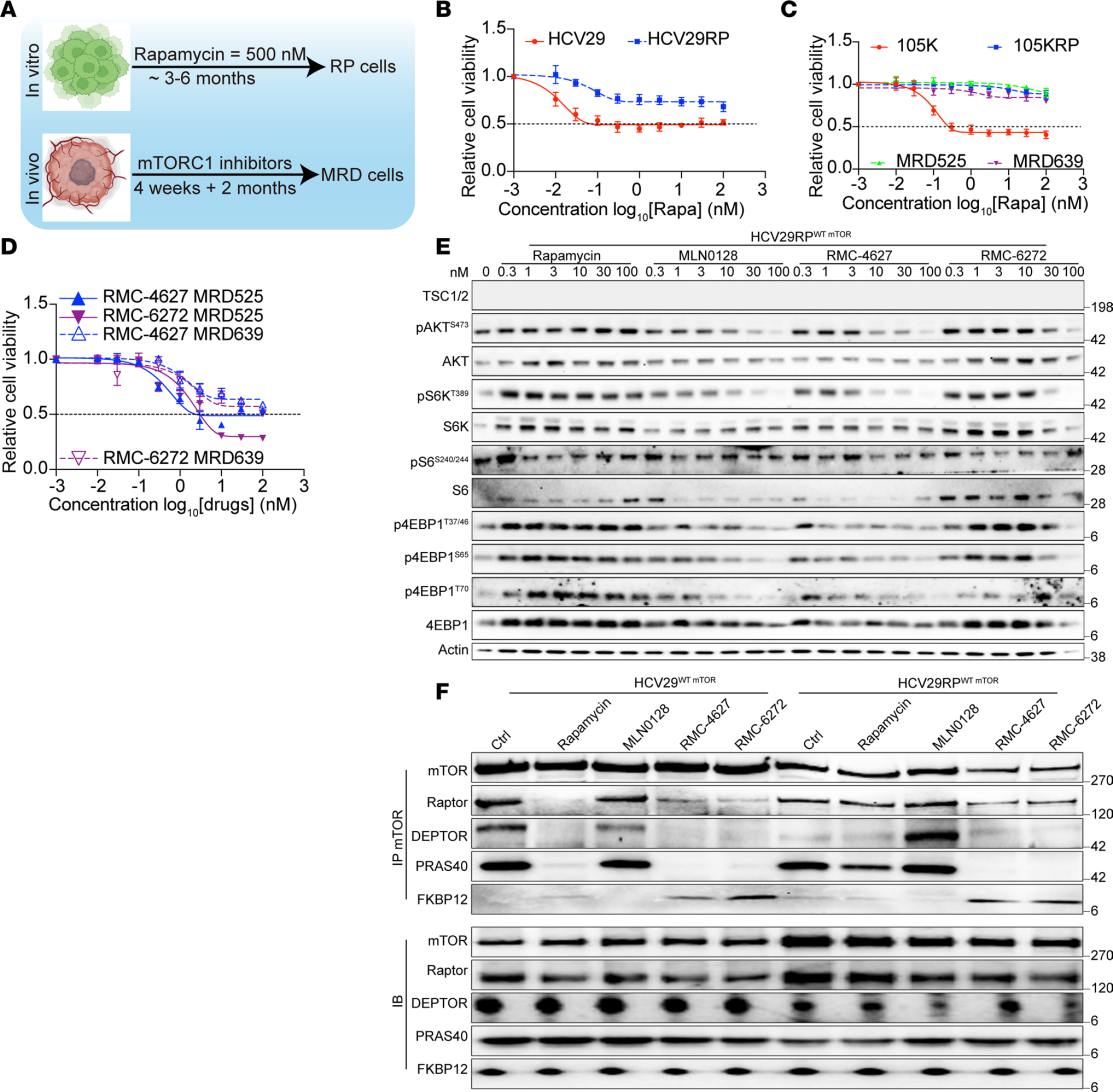
Long-term mTORC1 suppression gives rise to RP cells/MRD. (**A**) Schematic diagram showing the generation of RP and MRD models. (**B** and **C**) IC_50_ curve of rapamycin on RP (**B** and **C**) and MRD cells (**C**) and their parental counterpart cells. Each dot and error bar on the curves represent mean ± SD (*n* = 6). (**D**) IC_50_ curve for 2 MRD cell lines during treatment with bi-steric mTORC1 inhibitors. (**E**) Immunoblot of HCV29RP cells treated with different concentrations (nM) of mTORC1 inhibitors for 4 hours. Values on right are in kilodaltons. (**F**) mTOR co-IP blots of HCV29 and HCV29RP cells pretreated with different mTORC1 inhibitors (10 nM, 30 minutes).

Nine cell lines with mTORC1 hyperactivation secondary to TSC1/TSC2 loss from various organs/tissues (6 from human cancers) were exposed to 500 nM rapamycin for more than 3 months, leading to generation of RP cells (Supplemental Table 1; supplemental material available online with this article; https://doi.org/10.1172/jci.insight.187448DS1). MRD cells were also generated in vivo, by treating mouse xenografts of the 105K Tsc2^–/–^ RCC line in syngeneic C57BL/6J mice with rapamycin (3 mg/kg, intraperitoneally [IP] 3 times/wk for 1 month, MRD525), or the bi-steric inhibitor RMC-6272 (8 mg/kg, IP once/wk for 1 month, MRD639), followed by 2 months of treatment cessation (Figure 1A). In comparison with parental cells (17), all 9 RP cell lines showed little or no growth inhibitor effect from rapamycin (IC_50_ > 100 nM) and were variably sensitive to an mTORC1 bi-steric inhibitor (RMC-6272), with IC_50_ > 10 nM — not reached in comparison with an IC_50_ < 1 nM for these compounds in the starting lines (Figure 1, B–D, and Supplemental Figure 1, A–D). The bi-steric inhibitor–treated MRD line MRD639 had an IC_50_ > 100 nM for the 2 bi-steric inhibitors (Figure 1D and Supplemental Table 1).

**Figure 2:**
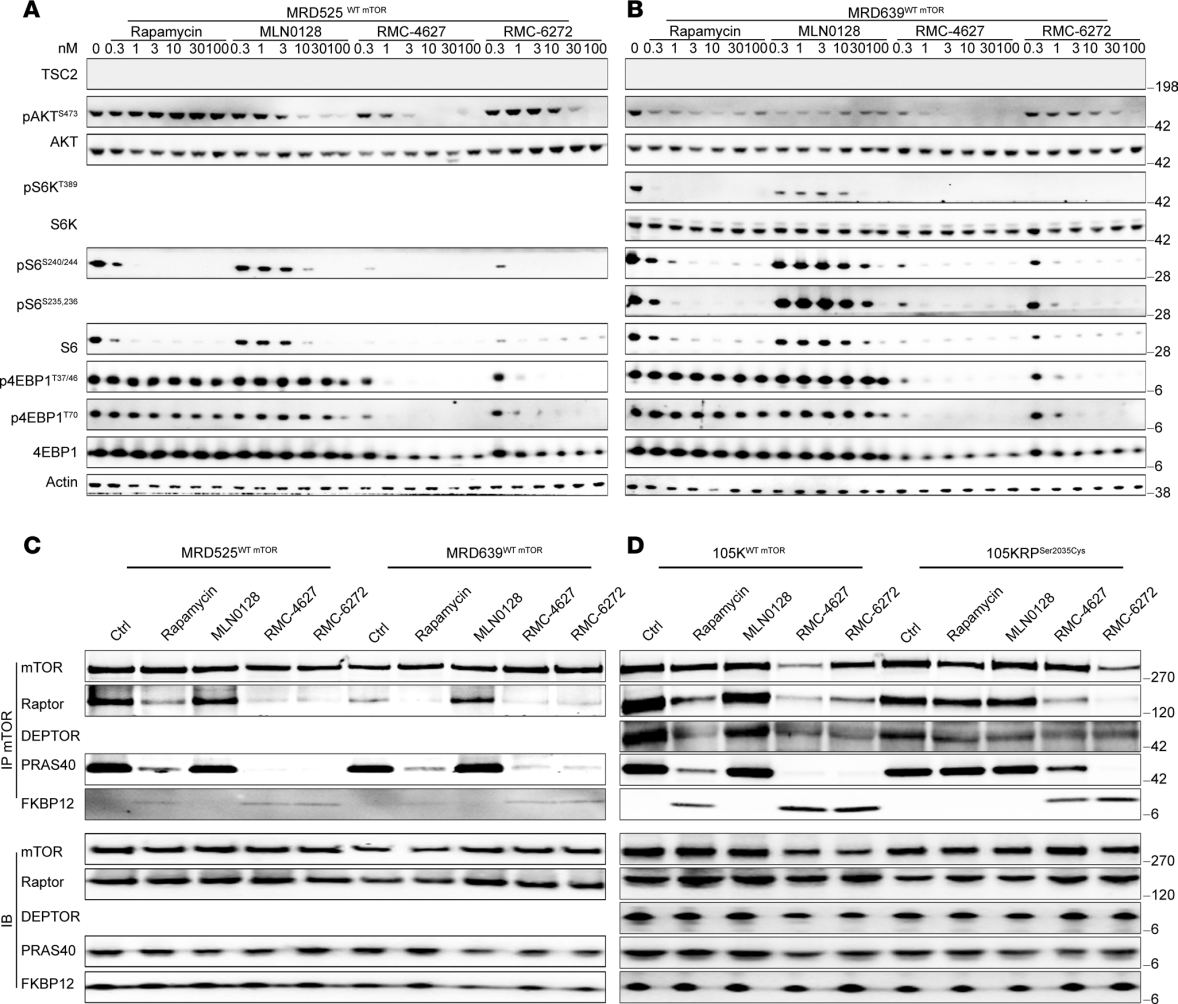
Characterization of mTORC1 signaling pathway of MRD developed in vivo. (**A** and **B**) Immunoblot of MRD525 (**A**) and MRD639 (**B**) cells treated with different concentrations (nM) of mTORC1 inhibitors for 4 hours. (**C** and **D**) mTOR co-IP blots of MRD525 and MRD639 (**C**), 105K, and 105KRP (**D**) cells pretreated with different mTORC1 inhibitors (10 nM, 30 minutes).

mTORC1 signaling pathway analysis showed that even 100 nM rapamycin had mild or no effect on pS6K^T389^, pS6^S235,236^, pS6^S240,244^, p4EBP1^T37,46^, p4EBP1^S65^, and p4EBP1^T70^, and those phosphorylation sites were less sensitive to bi-steric inhibitors in all 9 RP cell lines (Figure 1E; Supplemental Figure 1, E–K; and Supplemental Figure 2E). However, mTORC1 activity was dramatically decreased by both rapamycin and bi-steric inhibitors at 1 nM in MRD525 and MRD639 cells (Figure 2, A and B).

Whole-exome sequencing revealed that 3 mouse RP cell lines (mouse embryonic fibroblasts [MEFs] *Tsc2*^–/–^, 105K, m705) had acquired *Mtor* mutations at S2035 in the FKBP12-rapamycin-binding (FRB) domain, a site previously identified as being the key residue for rapamycin binding (32). The RP derivative of RT4, a human BLCA cell line, acquired mutations at both S2034 in the FRB domain and I2398 in the kinase domain (Supplemental Table 1). The remaining 3 RP cell lines that were sequenced showed no mutations in *MTOR*. Other genes encoding proteins involved in mTORC1 signaling, including *RAPTOR*, *PRAS40*, *DEPTOR*, *FKBP12*, *MLST8*, and *SIN1*, also did not have mutations in any of the 7 RP cells subjected to sequencing.

To examine how *MTOR* mutations affect rapamycin binding affinity, we performed immunoprecipitation of mTORC1 using anti-MTOR antibody followed by immunoblotting (Figure 1F; Figure 2, C and D; Supplemental Figure 2, A–D). *Mtor^Ser2035Cys^* (105KRP) eliminated binding of rapamycin-FKBP12 to MTOR in contrast with the starting cell line (Figure 2D), while binding of bi-steric inhibitors was not affected. Cells with *Mtor^Ser2035Phe^* (MEFs Tsc2-RP) showed reduced binding of both rapamycin and bi-steric inhibitors (Supplemental Figure 2B); cells with *Mtor^Ser2035Pro^* (m705RP) showed normal binding of rapamycin and bi-steric inhibitors-FKBP12 in this assay (Supplemental Figure 2C). The binding affinities of RP/MRD cells with WT MTOR showed no major change (Figure 1F, Figure 2C, and Supplemental Figure 2, A and D). RNA-Seq analysis of paired starting and RP sublines showed a range of expression of mTORC1 and mTORC2 components, but relatively little change in expression with induction of rapamycin persistence, with the exception of the SNU886RP line, in which FKBP12 expression was markedly reduced, likely contributing to the acquisition of resistance to rapamycin (Supplemental Figure 2F). Several MTOR mutations have been associated with resistance to rapamycin in human patients with cancer, including those affecting S2034 and S2035 (33) (Supplemental Figure 2G). However, 3/7 of our RP/MRD models had no mutations in MTOR (by whole-exome sequencing), or expression differences, suggesting that mTOR mutation–independent mechanisms are involved in their persistence to long-term mTORC1 suppression.

### Comprehensive reprogramming occurs in RP/MRD cells.

**Figure 3:**
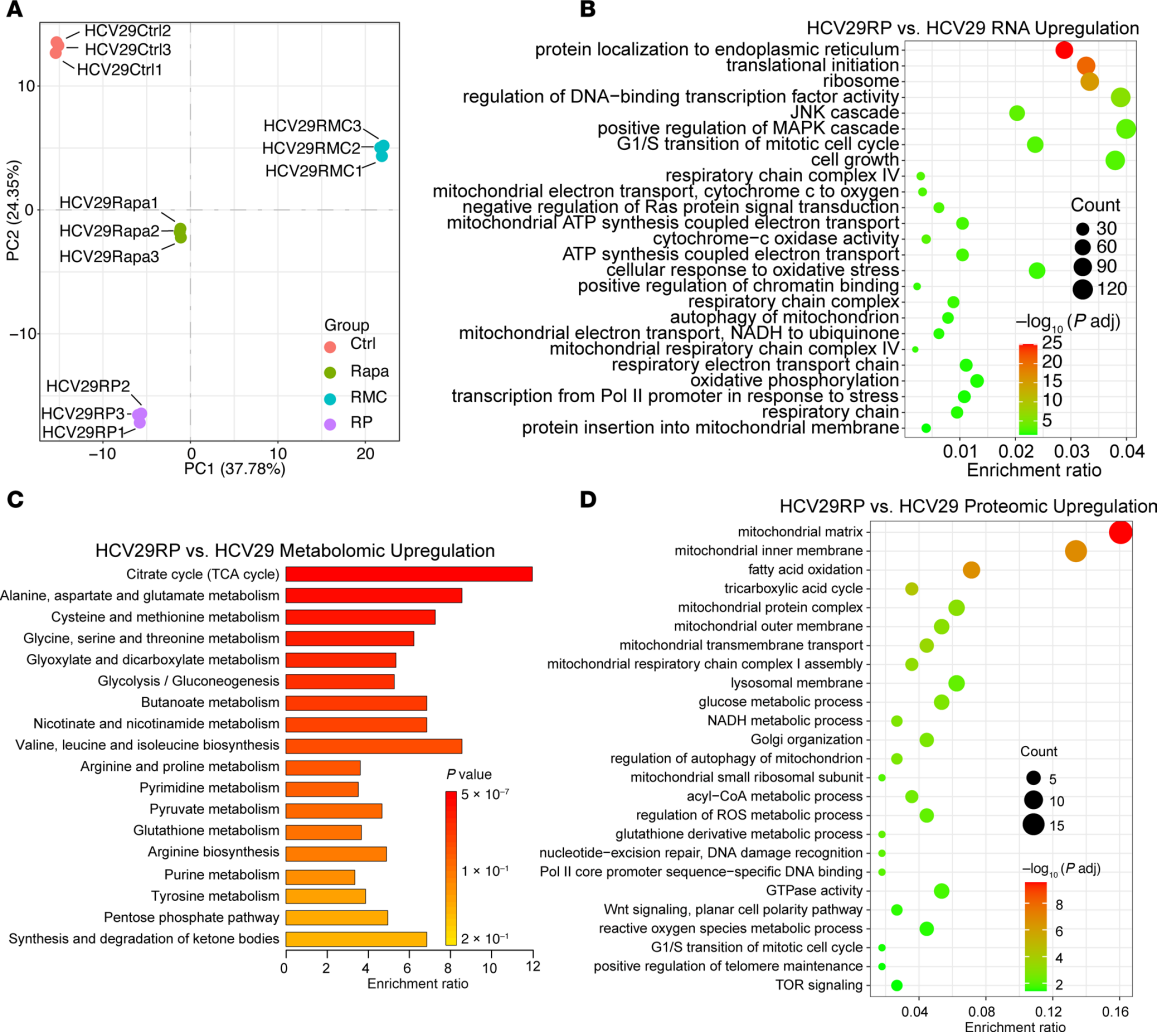
HCV29RP/MRD cells display metabolomic and proteomic differences that suggest enhancement of mitochondrial activity. (**A**) PCA plot of expression data for untreated, rapamycin-treated (10 nM, 24 hours), RMC-6272–treated (RMC-treated) (10 nM, 24 hours), and HCV29RP cells shows consistent expression differences. (**B**) GSEA of RNA-Seq shows enrichment of expression in genes involved in translation and ribosome in HCV29RP compared with HCV29 cells. (**C**) MSEA shows enrichment for TCA cycle metabolites in HCV29RP compared with HCV29 cells. (**D**) Proteomic set enrichment analysis shows enrichment for proteins in the mitochondria matrix and inner membrane in HCV29RP compared with HCV29 cells (*n* = 3 for each group).

To explore further the mechanism of persister development, comprehensive multiomic profiling was performed including RNA-Seq, metabolomics, proteomics, and phosphoproteomics on 2 pairs of cell lines. Principal component analysis (PCA) showed that HCV29RP and m705RP cells had a distinct transcriptomic profile compared with parental cells or parental cells treated with short-term (24 hours) rapamycin or the bi-steric RMC-6272 (Figure 3A and Supplemental Figure 3A). Gene set enrichment analysis (GSEA) identified many pathways enriched in genes with a change in expression in the RP cells, including translation initiation and ribosome, regulation of transcription factor activity, cell growth, and mitochondrial function (Figure 3B and Supplemental Figure 3B). Metabolomic set enrichment analysis (MSEA) identified the TCA cycle as the pathway with the most changes in metabolite levels in HCV29RP (Figure 3C). Proteomic analysis also identified mitochondrial and OXPHOS-related pathways as those having proteins with the greatest enrichment in peptide counts by mass spectrometry in HCV29RP cells (Figure 3D). Similar results were obtained in 2 other RP cell lines, as shown by RNA-Seq analysis (m705RP, H101RP, Supplemental Figure 3, B–F).

Our previous study exhibited that low-concentration (10 nM) and short-term (within 1 hour) rapamycin or RMC-6272 affects the phosphorylation levels of multiple targets within the PI3K/AKT/mTOR pathway. Global phosphoproteomic analysis showed that proteins involved in ribonucleoprotein (RNP) biogenesis, RNA processing, and ribosome biogenesis were the proteins whose phosphorylation was most commonly downregulated in short-term rapamycin- or RMC-6272–treated m705 cells (17). In contrast, m705RP cells had dramatically increased phosphorylation levels of proteins participating in RNA processing and splicing, RNP synthesis, ribosome biogenesis, and chromatin assembly and remodeling, in comparison with m705 cells (Supplemental Figure 3E). Hence, long-term rapamycin treatment induces an expression, metabolic, and proteomic state that is very different from the acute effects of rapamycin on mTORC1 inhibition. Furthermore, this occurs in cell lines both with and without *MTOR/Mtor* mutation.

### Long-term mTORC1 suppression rewires glycolysis to OXPHOS.

**Figure 4:**
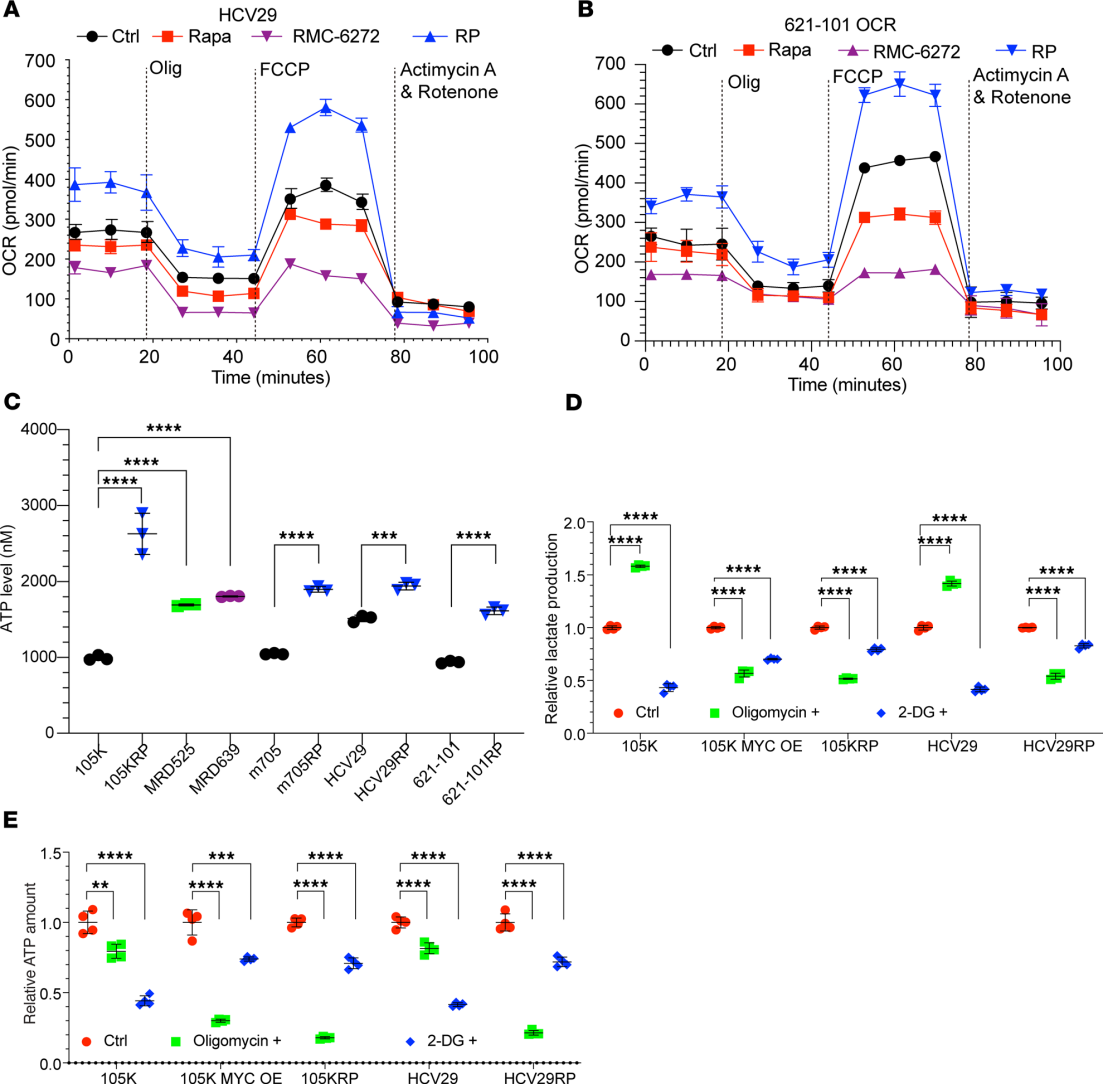
RP cell lines have upregulated OXPHOS. (**A**) Seahorse assay data for untreated, rapamycin-treated (10 nM, 24 hours), and RMC-6272–treated (10 nM, 24 hours) HCV29 cells (parental cells), and untreated HCV29RP cells shows an increase in OCR in the RP cells following both oligomycin and carbonyl cyanide *p*-trifluoromethoxyphenylhydrazone (FCCP) treatment. RMC-6272 shows a greater reduction in OCR than rapamycin under all conditions. (**B**) Similar data for 621-101 cells. (**C**) ATP levels in cell lysates from multiple control/starting cell lines and RP or MRD derivatives. (**D** and **E**) Lactate and ATP levels in parental, MYC overexpression (OE), and RP cell lines (*n* = 3 or *n* = 4). 2-DG, 2-deoxy-d-glucose. Each dot and error bar on the curves represent mean ± SD (*n* = 3). One-way ANOVA was used. **P < 0.01, ***P < 0.001, ****P < 0.0001.

**Figure 5:**
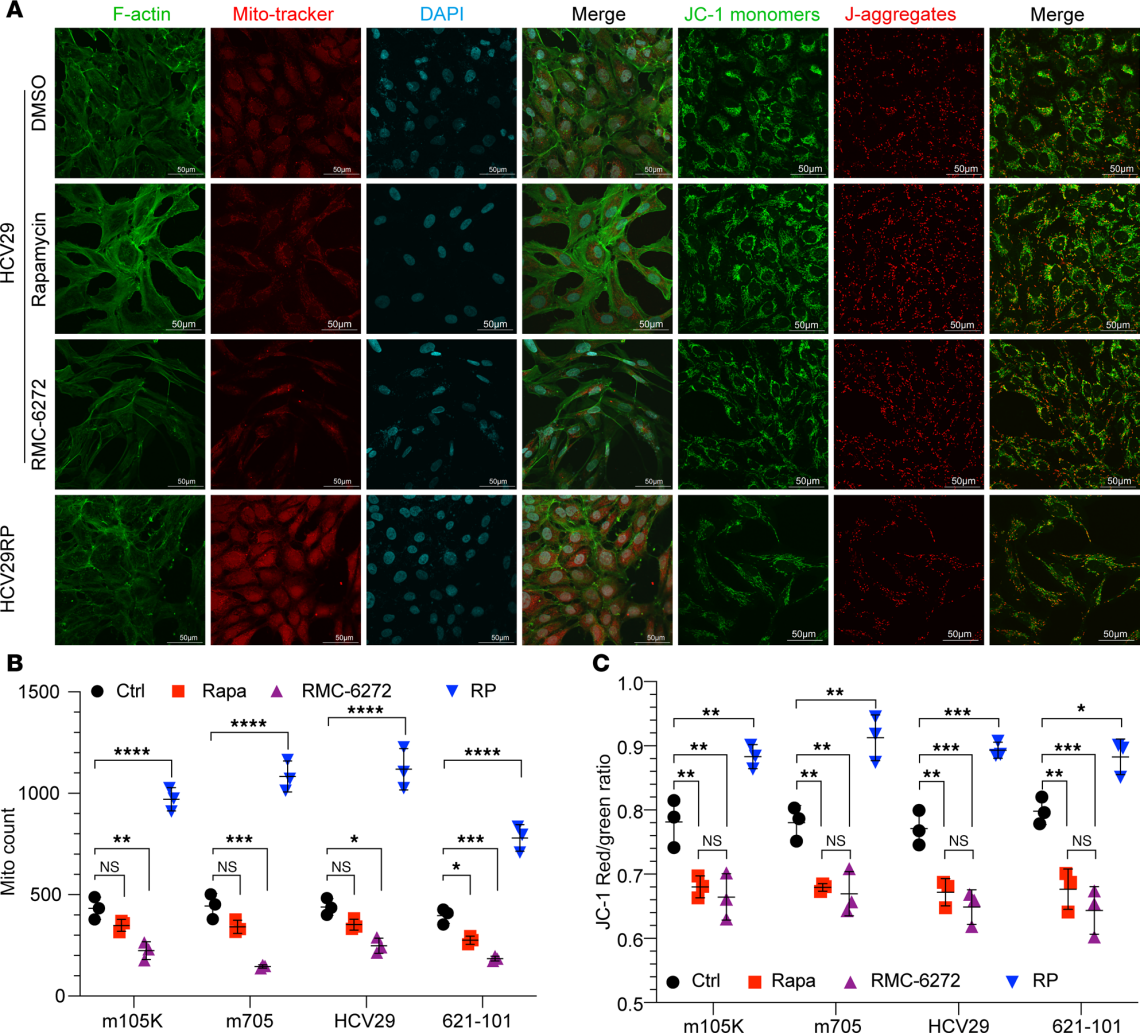
RP cells have increased numbers of mitochondria. (**A**–**C**) RP cells have increased mitochondrial numbers as measured by MitoTracker and JC-1 staining (**A**) followed by quantification (**B** and **C**). Each dot and error bar on the curves represent mean ± SD (*n* = 3). One-way ANOVA was used. *P < 0.05, **P < 0.01, ***P < 0.001, ****P < 0.0001.

Two observations led us to examine OXPHOS in greater detail in the RP cells. First, the enhancement in apparent mitochondrial activity from the -omic analysis above, and second, a reversal in the classic glycolytic state induced by loss of either TSC1 or TSC2, and mTORC1 activation. The media of RP/MRD cells showed a lack of acidification (media stayed red) in contrast with their parental counterpart cells, in which the media turned yellow within 2–3 days. The Seahorse assay was performed comparing multiple pairs of RP versus parental cell lines. HCV29RP and H101RP cells had an increased oxygen consumption rate (OCR) at baseline in comparison with parental cells. mTORC1 inhibition by rapamycin or the bi-steric inhibitor RMC-6272 (24 hours) in parental cells decreased OCR (Figure 4, A and B). Similar results were seen for the m705RP and 105KRP cells (Supplemental Figure 4, A and B). Consistent with this finding, multiple RP/MRD cell lines had higher ATP levels than their starting cells (Figure 4C). MitoTracker and JC-1 staining followed by quantification showed that short-term mTORC1 suppression, especially by RMC-6272, significantly decreased mitochondrial membrane potential, whereas RP/MRD cells showed a significant increase in mitochondrial membrane potential and number of mitochondria per cell (Figure 5, A–C, and Supplemental Figure 4C). RP/MRD cells also showed upregulated ROS- and oxidative stress–related pathways by multiomics (Figure 3, B and D, and Supplemental Figure 3, B, D, and G) and increased reducing activity, including glutathione metabolism (Figure 3C and Supplemental Figure 3C), consistent with an increase in ROS generation due to enhanced OXPHOS. Recurrent MRD tumors after mTORC1 inhibitor treatment cessation also showed increased mitochondria staining. There was a consistent increase (about 2-fold) in mitochondria number and the OCR and mitochondrial membrane potential, assessed using JC-1, in RP/MRD cells compared with parental cells, indicating that the RP/MRD cells had increased mitochondria numbers rather than enhanced mitochondrial function (Figure 3B, Figure 4A, and Figure 5B). To further validate a switch from glycolysis to OXPHOS in RP/MRD cells, oligomycin treatment increased media lactate levels in the starting lines but had the reverse effect on the RP lines (Figure 4D). The glycolysis inhibitor 2-DG reduced lactate generation in parental cells (~50%) with a milder effect on the RP cells (decreased ~25%, Figure 4D). Oligomycin treatment markedly reduced ATP levels (80%–90% reduction) in RP cells, while it had a much milder effect on the parental cells (~20% reduction), while 2-DG treatment had the opposite effect (Figure 4E). These observations indicate that RP cells have an impaired glycolytic capacity and have made a switch from glycolysis to OXPHOS.

### MYC drives enhanced mitochondrial function and OXPHOS.

**Figure 6:**
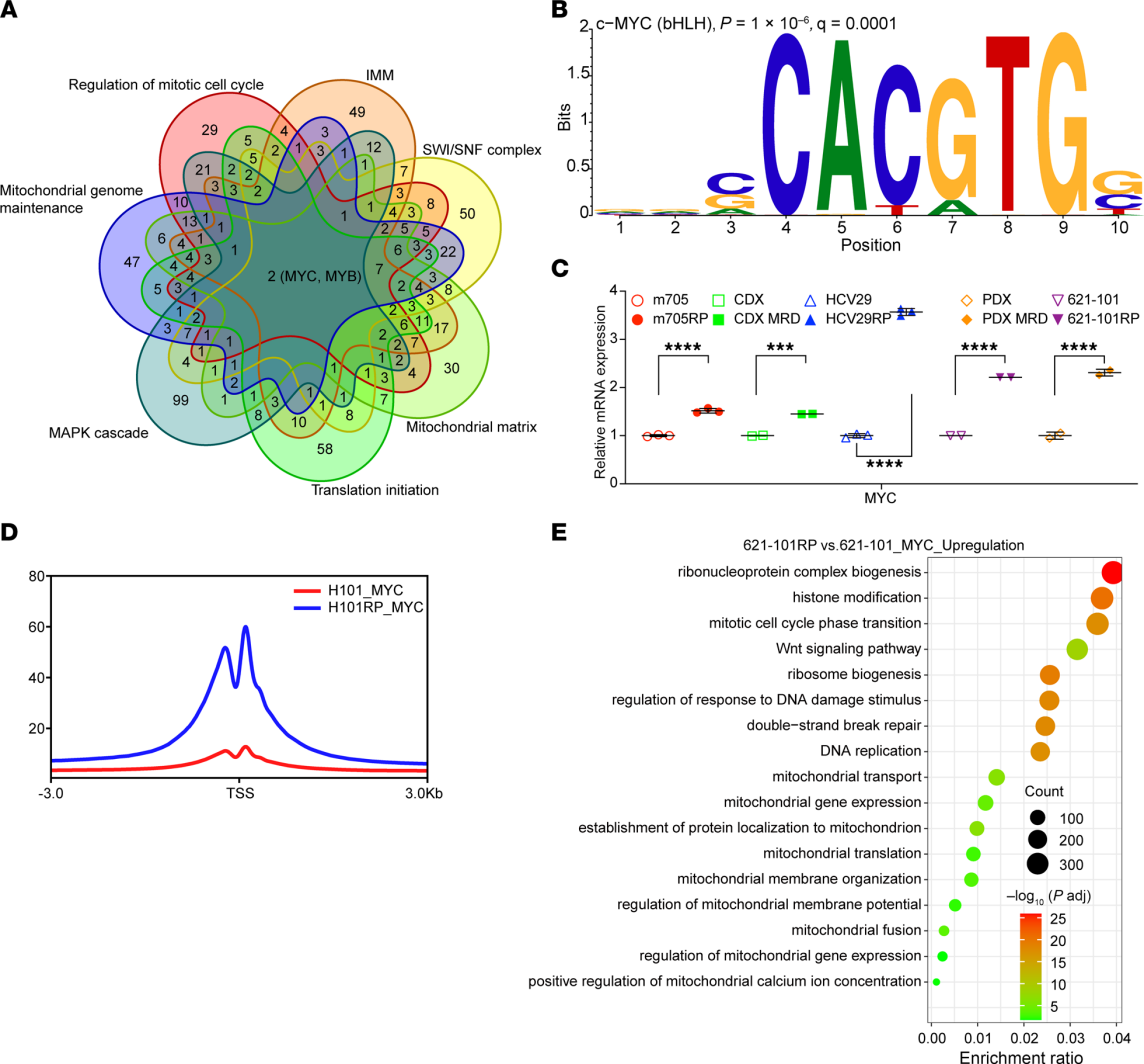
MYC is the TF that drives RP phenotypes. (**A**) Venn diagram shows the potential TFs that regulate the commonly and significantly upregulated pathways in RP cells compared with their parental cells. MYB and MYC are the 2 predicted TFs that overlapped in all 7 pathways. IMM, inner mitochondrial membrane. (**B**) MYC motif is the most enriched motif found by HOMER using the promoter regions (defined as TSS ± 2,000 bp) of all the genes obtained in **A**. (**C**) Relative MYC mRNA expression level of multiple RP/MRD versus parental counterpart cells (*n* = 2 or *n* = 3). Each dot and error bar on the curves represent mean ± SD (*n* = 3). A 2-tailed Student’s *t* test was used. ***P < 0.001, ****P < 0.0001. (**D**) Profiling plot of MYC CUT&RUN of 621-101RP versus 621-101. (**E**) Pathway enrichment analysis using the differential peaks obtained from MYC CUT&RUN.

The induction of enhanced mitochondria respiration was consistent among the RP/MRD cells, and was independent of MTOR or other mutations, as above. Therefore, we hypothesized that an epigenetic event had occurred in RP/MRD cells during selection by inhibiting mTORC1 activation. The most commonly upregulated pathways in RP cells were selected to predict the potential transcription factors (TFs) regulating the genes within each pathway. The top 200 potential TFs of each pathway were intersected to obtain the TFs that could cover all the pathways. MYC and MYB were the 2 TFs that overlapped among all the selected pathways (Figure 6A). This result was further validated by motif analysis using the promoter regions (defined as TSS ± 1,500 bp) of all the commonly upregulated genes of RP/MRD cells (Figure 6B). RNA-Seq showed that MYC expression was 1.5–4 times higher in RP/MRD cells than parental cells (Figure 6C). To examine open chromatin globally, we performed histone protein H3 with acetylation of lysine 27 residue (H3K27ac) CUT&RUN, followed by Rank Ordering of Super Enhancers (ROSE), and observed that while MYC was not a superenhancer in m705 cells, it had acquired that status in m705RP cells (Supplemental Figure 5A). MYC CUT&RUN showed that RP cells had higher signals around TSS regions (Figure 6D and Supplemental Figure 5B). Genome regions with differential MYC binding were identified (Supplemental Figure 5C) followed by gene assignment and pathway enrichment analysis. Genes in pathways involved in RNP biogenesis, histone modification, cell cycle and replication, and mitochondrial function showed the greatest increase in MYC binding in RP cells compared with parental cells (Figure 6E and Supplemental Figure 5D). Taken together, these data suggest that enhancement of MYC expression and transcriptional activity contributes in a major way to continued growth and enhanced mitochondrial metabolism in the RP cells.

### SWI/SNF and MYC cooperate with each other in RP/MRD cells.

**Figure 7:**
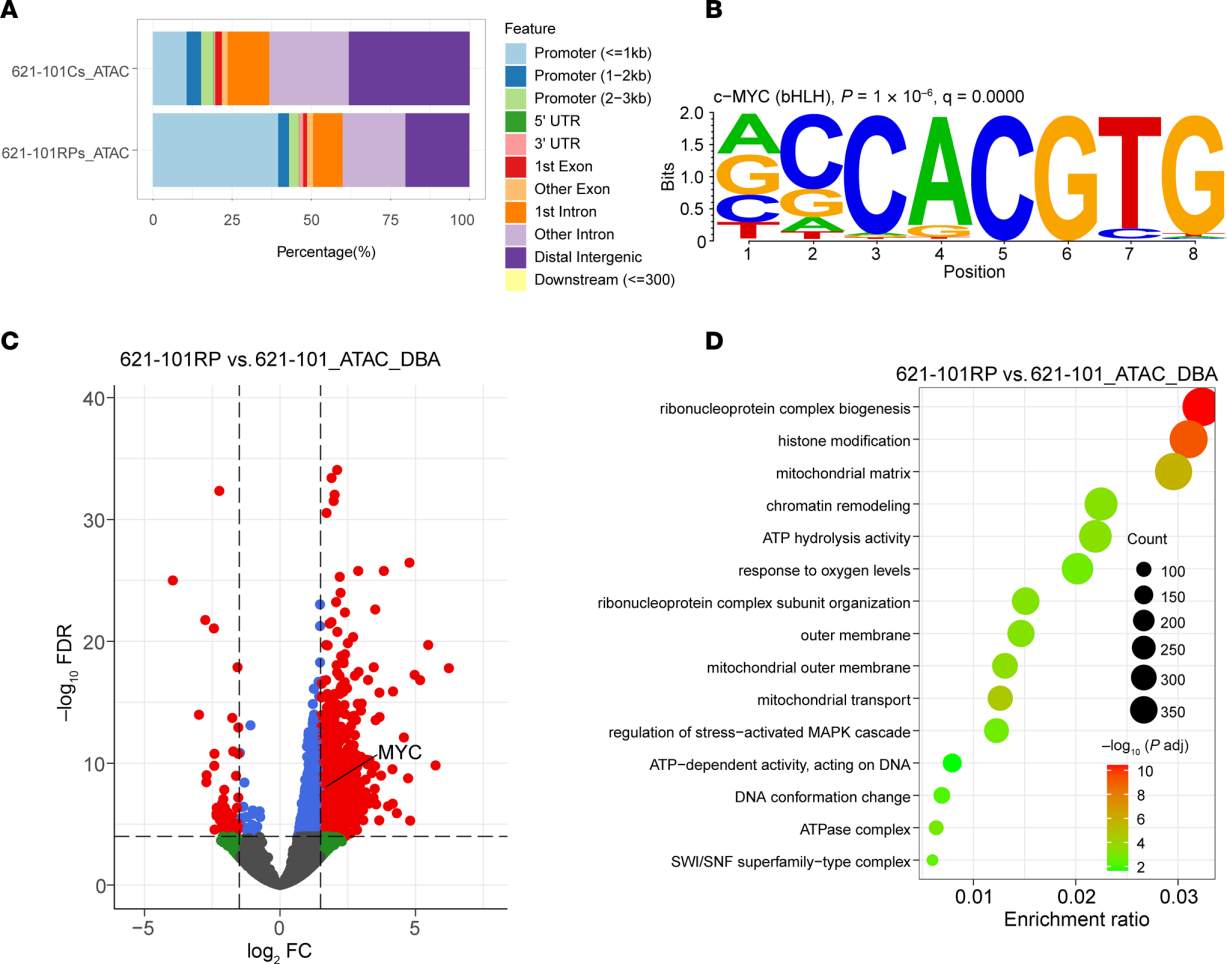
RP cells have increased chromatin accessibility. (**A**) Peak distribution of ATAC-Seq comparing 621-101RP versus 621-101 (*n* = 2). (**B**) Motif analysis of 621-101RP MYC CUT&RUN (*n* = 2). (**C**) Volcano plot shows genes with different accessibilities as judged by ATAC-Seq (*n* = 2). Gray indicates those genes with |log_2_ fold change| < 1.5 and not statistically significant; green indicates those genes with |log_2_ fold change| > 1.5 and not statistically significant; blue indicates those genes with |log_2_ fold change| < 1.5, and padj < 0.0001; and red indicates those genes with |log_2_ fold change| > 1.5, and padj < 0.0001. (**D**) Pathway enrichment analysis using the differential peaks obtained from ATAC-Seq.

Multiomic and MYC CUT&RUN analysis found that chromatin/histone modification/remodeling pathways were also enriched in RP/MRD cells (Figure 3B, Supplemental Figure 3E, and Figure 4F). We hypothesized that SWI/SNF, an ATP-dependent chromatin remodeler, might be involved in the chromatin remodeling in RP cells, given increased OXPHOS and higher ATP levels in RP cells (Figures 4 and 5). To explore this possibility, we performed the assay for transposase-accessible chromatin with sequencing (ATAC-Seq) on the RP cells. Interestingly, more open chromatin regions defined by ATAC-Seq were enriched in promoter regions in RP cells compared with parental cells (Figure 7A and Supplemental Figure 5F). Motif analysis of ATAC-Seq peaks showed that the MYC motif was significantly enriched in RP cells but not in parental cells (Figure 7B). Differential peak analysis followed by gene annotation identified many genes with a marked change in ATAC-Seq signal, including MYC, which had increased chromatin accessibility in RP cells (Log_2_FC = 1.52, FDR = 2.04 × 10^–8^; Figure 7C; Log_2_FC = 4.42, FDR = 0, Supplemental Figure 5E). Furthermore, pathway analysis of differentially open ATAC-Seq regions identified RNP complex formation, histone modification, mitochondrial, and chromatin remodeling pathways (Figure 7D and Supplemental Figure 5G), consistent with the multiomic and H3K27ac CUT&RUN findings.

**Figure 8:**
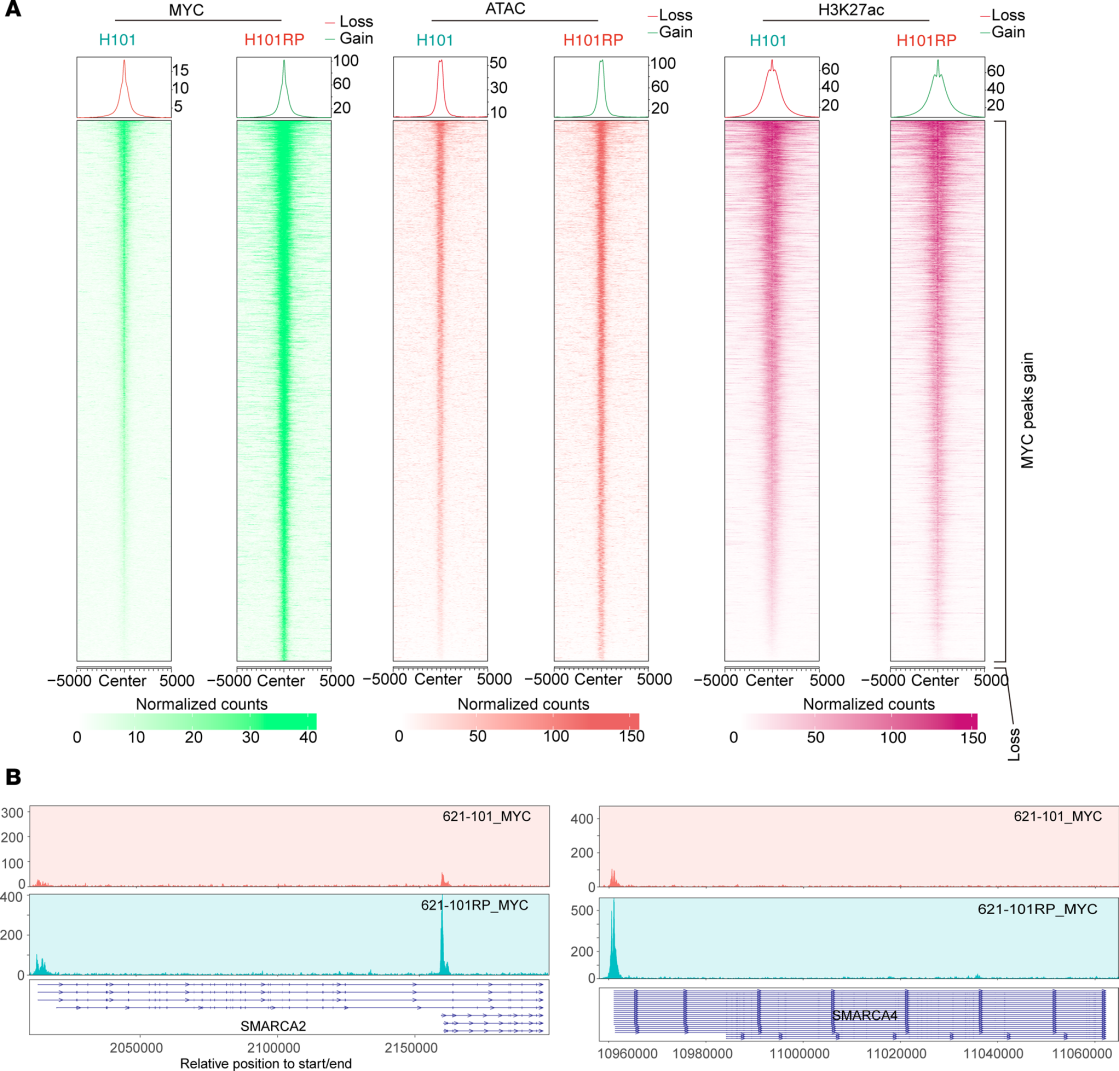
Cooperation of SWI/SNF and MYC reshapes chromatin landscapes in RP cells. (**A**) Profiling heatmap of MYC CUT&RUN, H3K27ac CUT&RUN, and ATAC-Seq, aligned to the differentially expressed MYC CUT&RUN peaks. (**B**) MYC binding at TSS of SMARCA2 and SMARCA4 in 621-101RP compared with 621-101 cells.

**Figure 9:**
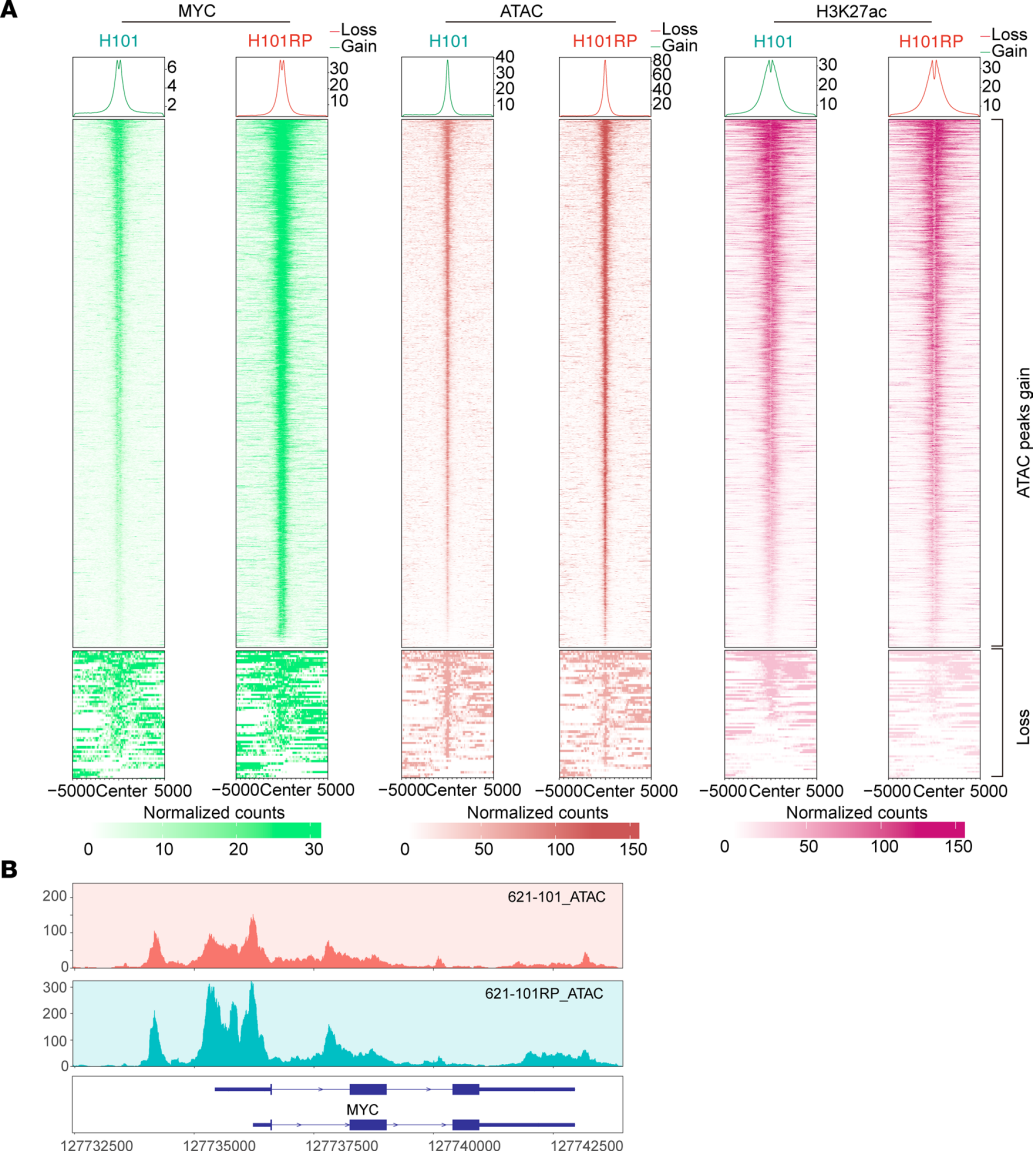
Increased chromatin accessibility facilitates MYC binding in RP cells. (**A**) Profiling heatmap of MYC, H3K27ac, CUT&RUN, and ATAC-Seq, aligned to the differentially expressed ATAC-Seq peaks. (**B**) ATAC-Seq signal showing more open chromatin at MYC gene in 621-101RP compared with 621-101 cells.

**Figure 10:**
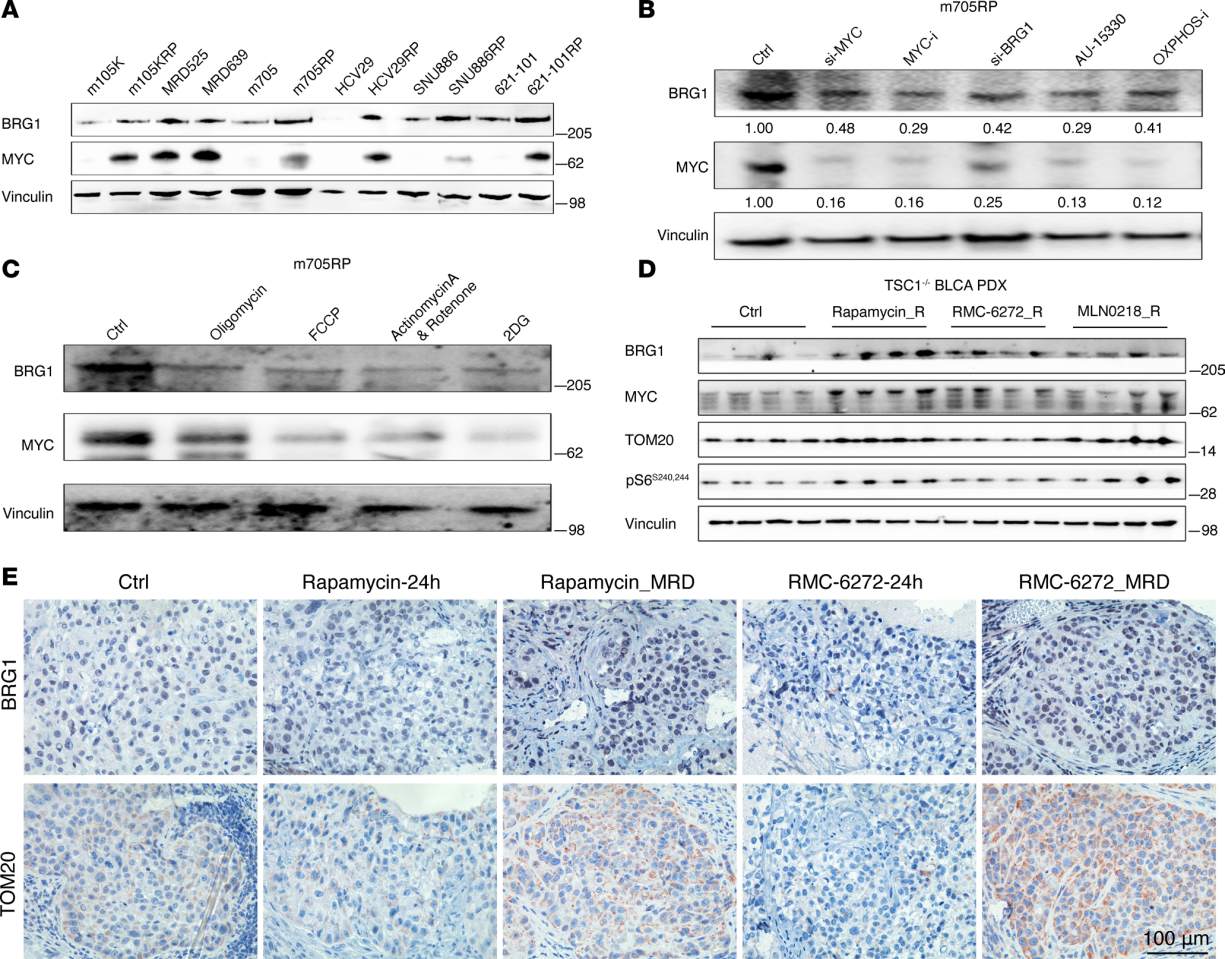
MRD tumors developed in vivo exhibit upregulated MYC and BRG1. (**A**) BRG1 and MYC protein expression in multiple RP/MRD versus parental counterpart cells. (**B** and **C**) BRG1 and MYC expression after suppression of BRG1, MYC, OXPHOS, or mitochondria function. (**D**) BRG1, MYC, and TOM20 expression in PDX_MRD tumors developed after the 1-month treatment of vehicle or different mTORC1 inhibitors followed by 2-month treatment cessation. si, siRNA. (**E**) Staining of mitochondria using TOM20 and BRG1 in BLCA tumors after 24 hours of rapamycin (3 mg/kg, i.p., dosing once) or RMC-6272 (8 mg/kg, i.p., dosing once) treatment and in recurrent tumors 2 months after 1 month of treatment with rapamycin (3 mg/kg, i.p., 1 month) or RMC-6272 (8 mg/kg, i.p., 1 month). TOM20, mitochondrial import receptor subunit TOM20 homolog; PDX, patient-derived xenograft.

Comparison of MYC and H3K27ac CUT&RUN and ATAC-Seq signals at genomic regions with enhanced MYC binding showed correlation among them (Figure 8, A and B, and Supplemental Figure 6, A and B). Correlation was also seen for the peaks for which the ATAC-Seq signal was enhanced in the RP cells (Figure 9, A and B). Interestingly, RP cells had much stronger MYC binding in the TSS regions of SMARCA2 and SMARCA4, compared with parental cells (Figure 8B and Supplemental Figure 6, C and D), while MYC had a much stronger ATAC-Seq signal (Figure 9B and Supplemental Figure 6E). Expression of both BRG1 (SMARCA4 product) and MYC protein was increased in multiple RP/MRD cells in comparison with parental cells (Figure 10A). For MYC this increase in protein levels was dramatic, and much greater than the change in MYC RNA levels (Figure 6C), suggesting that translation or posttranslation modification of MYC contributed, which was consistent with the increased translation-related pathways from RNA-Seq analysis (Figure 3B). MYC suppression by KD or MYC inhibitor decreased BRG1 expression (Figure 10B). MYC and BRG1 protein expression was decreased when OXPHOS was inhibited with IACS-010759 and when SWI/SNF was inhibited with AU-15330 (Figure 10B). In addition, treatment with the mitochondrial uncoupler FCCP, oligomycin, or glycolysis inhibitor (2-DG) led to decreased MYC and BRG1 protein expression (Figure 10C) and decreased SWI/SNF activity (as assessed by chromatin accessibility by ATAC–quantitative PCR [ATAC-qPCR], Supplemental Figure 7C).

To further explore the potential “cooperation” between MYC and SWI/SNF and their individual effects, MYC was overexpressed (stable OE by lentivirus infection) in the parental cells (Supplemental Figure 7A). Parental cells with MYC OE became resistant to rapamycin even at 100 nM, mimicking the 105KRP, MRD525, or MRD639 (Figure 1C and Supplemental Figure 7B). Furthermore, MYC OE cells had a similar SWI/SNF activity in comparison to the 105KRP cells, as measured by ATAC-qPCR (Supplemental Figure 7C). Glycolysis/Oxidative phosphorylation assay showed that the MYC OE cells had a metabolic switch from glycolysis to OXPHOS (Figure 4, D and E), which led to upregulated ROS generation and higher level of lipid peroxidation (Supplemental Figure 7, D and E). Thus, MYC OE reproduced in part the phenotype of RP cells.

To examine this effect in vivo, we used a previously published human BLCA PDX model, which had been treated with rapamycin for a month and then regrew (17). The regrown PDX had approximately 2.5-fold higher MYC mRNA levels (Figure 6C), and immunoblot and IHC staining showed higher levels of BRG1, MYC, and TOM20 (Figure 10, D and E). Differentially expressed gene followed by pathway enrichment analysis showed that mitochondrial, OXPHOS, translation, and chromatin remodeling genes/pathways were upregulated and enriched in the PDX_MRD tumors. Motif analysis of the promoter regions of those genes showed that MYC was the potential driver gene (Supplemental Figure 7, G and H).

Hence, these data suggest that MYC and SWI/SNF cooperate with each other in the chromatin rewiring of RP/MRD cells and highlight the critical role of MYC OE in this process.

### Combined inhibition of SWI/SNF or mitochondria complex I and mTORC1 inhibitors causes synergistic lethality by inducing cuproptosis.

**Figure 11:**
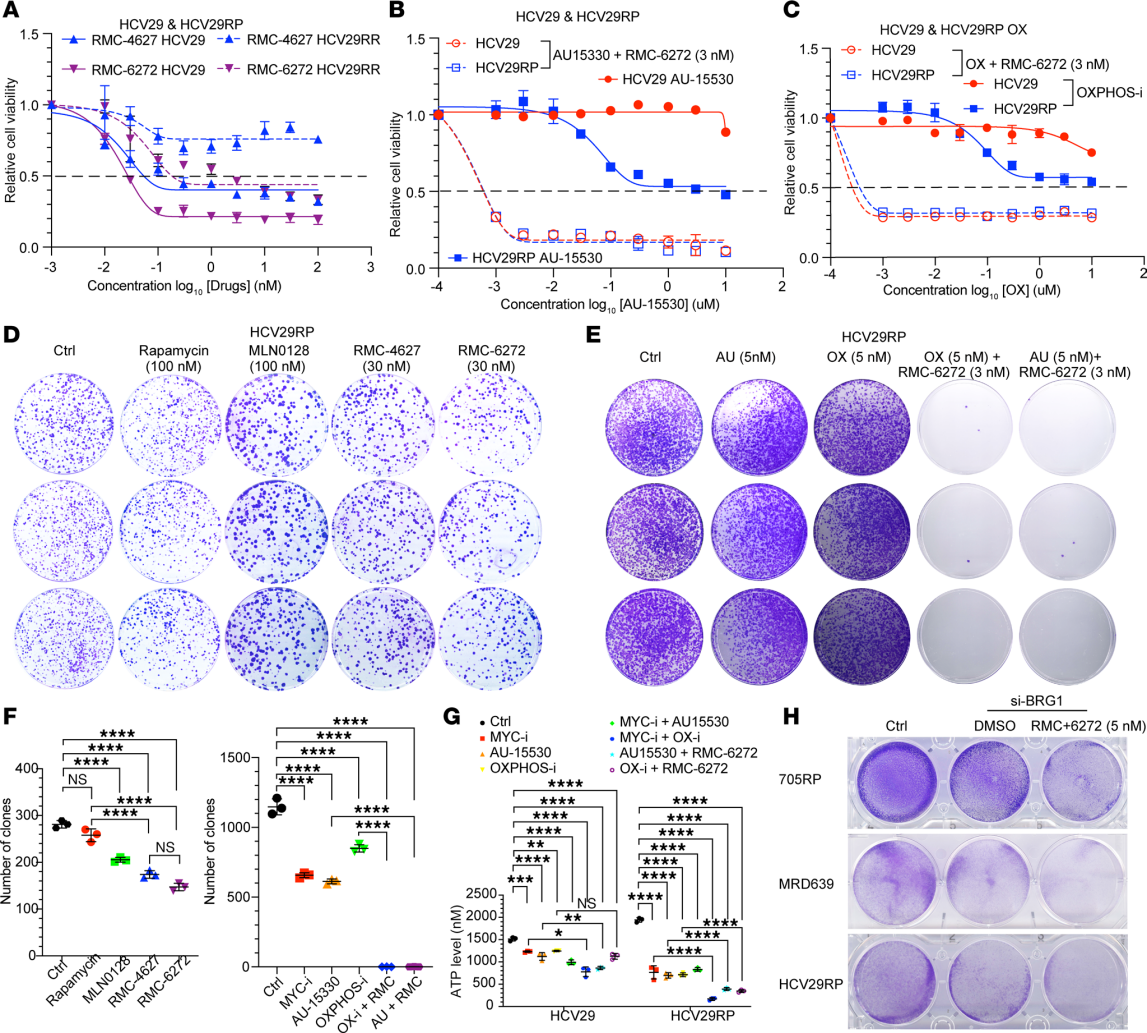
Inhibition of SWI/SNF function or OXPHOS shows synergy with bi-steric mTORC1-selective inhibitors in RP/MRD cells. (**A**–**C**) Dose-dependent cell growth inhibition curves of HCV29 and HCV29RP cells. Each dot and error bar on the curves represent mean ± SD (*n* = 6) in (**B** and **C**). Open circles are cells treated with fixed dose of 3 nM RMC-6272. (**D**–**F**) Low-dilution clone formation and clone number quantification of HCV29RP cells treated by different inhibitors. (**G**) ATP levels after different treatment in HCV29 and HCV29RP cells (24 hours). Each dot and error bar on the curves represent mean ± SD (*n* = 3). One-way ANOVA was used. *P < 0.05, **P < 0.01, ***P < 0.001, ****P < 0.0001. (**H**) BRG1 KD in multiple cell lines with or without RMC treatment.

As noted earlier, bi-steric mTORC1 inhibitors, which have very high binding affinity for mTORC1, had reduced but variably retained effects on RP/MRD cell proliferation in comparison with parental cells (Figure 11A; Supplemental Figure 8, A–G; and Supplemental Figure 9, A–J).

Since our studies above indicated that the SWI/SNF complex and mitochondrial activity were enhanced in RP/MRD cells, we examined the sensitivity of RP cells to inhibitors of SWI/SNF and OXPHOS. AU-15330, a proteolysis-targeting chimera degrader of the SWI/SNF ATPase subunits SMARCA2 and SMARCA4, and IACS-010759 (a mitochondrial complex I inhibitor) both showed differential effects on RP/MRD cell growth, in comparison with parental cells (Figure 10C, Figure 11B, and Supplemental Figure 8, B–J). Furthermore, AU-15330 and IACS-010759 showed strong synergy with RMC-6272 when used on either RP or the starting cells (Figure 11, B–F; Supplemental Figure 8, B, D, F, H, and J; and Supplemental Figure 9, K and L). Also, 3 nM RMC-6272 plus 5 nM AU-15330 or IACS-010759 led to dramatic depletion of ATP levels (Figure 11G). To confirm that the mechanism of action of IACS-010759 occurred due to its effects on expression of BRG1, we found that BRG1 siRNA combined with 5 nM RMC-6272 also had a dramatic effect on cell proliferation (Figure 11H).

**Figure 12:**
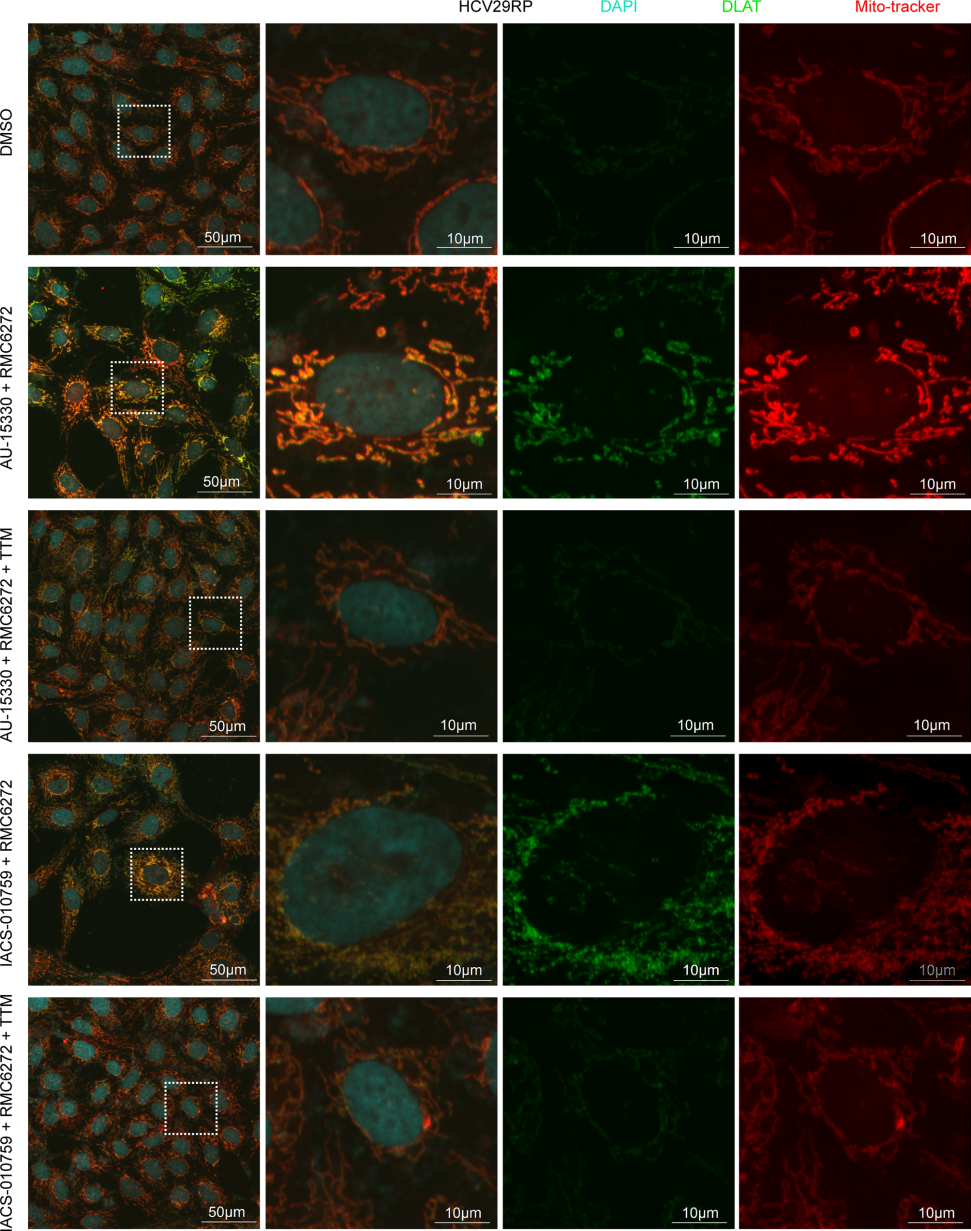
RP cells are hypersensitive to cuproptosis induction by combination of SWI/SNF inhibitor and RMC-6272. Confocal immunofluorescence imaging of DLAT (green) and MitoTracker (red) in HCV29RP cells treated with AU-15330 + RMC. TTM, tetrathiomolybdate.

It is known that cells that are more reliant on OXPHOS are more sensitive to cuproptosis (copper-induced cell death) (34). Given that RP/MRD cells rely more heavily on mitochondrial respiration than glycolysis in comparison with parental cells (Figure 6E, Supplemental Figure 3D, and Supplemental Figure 5, D and G), we hypothesized that RP/MRD cells were prone to cuproptosis. RP/MRD and parental cells were treated with different concentrations of elesclomol (copper ionophore, ES) and CuCl_2_ (ES+Cu, 1:1 ratio) for 3 days followed by cell viability measurement. The ES+Cu IC_50_ of RP/MRD cells was ~1 nM, which was 30–100 times lower than the parental cells (IC_50_ ~100–1,000 nM) (Supplemental Figure 10, A–D). To further validate the findings in vivo, we showed that the PDX_MRD tumors after rapamycin or RMC-6272 treatment cessation showed higher level of FDX1 and lipoic acid, which are important in regulating cuproptosis (Supplemental Figure 10E). Furthermore, treatment of RP/MRD cells with AU-15330 plus RMC-6272, or IACS-010759 plus RMC-6272, induced cuproptosis, as judged by DLAT staining (Figure 12). Chelation of copper with TTM or KD of FDX1 rescued cell death induced by AU-15330 plus RMC-6272 (Figure 12 and Supplemental Figure 10F). In contrast, AU-15330 or RMC-6272 treatment alone did not cause cuproptosis (Supplemental Figure 10F).

To further validate that RP cells are more sensitive to copper-induced cell death, we examined ROS levels in parental cells, RP cells, and MRD cells. RP and MRD cells had higher levels (>1.5-fold increase) of ROS compared with parental cells (Supplemental Figure 7D). Malondialdehyde levels, a measure of lipid peroxidation, were also increased in RP cells (~2-fold, Supplemental Figure 7E). Copper ion levels were also increased relative to baseline in both RP and MRD cells (~1.5-fold, Supplemental Figure 7F).

Having observed that concurrent blockade of mitochondrial complex I by IACS-010759 and RMC-6272 induced cell death, we assessed the effect of other mitochondrial complex inhibitors. Mitochondrial complex II (TTFA), III (antimycin A), and IV (oligomycin) inhibitors all showed synergy with concurrent RMC-6272 (Supplemental Figure 10, G–I).

## Discussion

Although well established as a key growth-supporting pathway for both normal and tumor cells, the first and the second generations of mTORC1 inhibitors have shown only modest impact in many human cancers (35). Early clinical trials investigating the activity of first-generation rapalogs (rapamycin, everolimus, temsirolimus, etc.) showed that these drugs typically caused modest tumor regression, with rare partial responses (PRs), and no complete remissions for RCC or pancreatic neuroendocrine tumors (36, 37). Rapalogs have also been used in combination therapy for breast cancer (38, 39). Greater and more consistent activity has been seen with these agents for treatment of the TSC syndromic tumors: renal AML, cardiac rhabdomyoma, LAM, and subependymal giant cell astrocytoma (15, 40–43). However, for these tumors as well, only occasional PRs by standard Response Evaluation Criteria in Solid Tumors criteria are seen, and long-term continuous therapy is required to maintain benefit, since tumor regrowth occurs upon treatment cessation (15, 40–43).

Recently, greater clinical benefit has been seen with the use of albumin-bound sirolimus (nab-sirolimus, FYARRO) in the treatment of PEComa: 39% of patients had PRs, with median duration of response of 40 months, and there was a 7% complete response rate (including a patient with surgical resection of residual disease) (44). In addition, bi-steric mTORC1 inhibitors have shown promise in preclinical mouse models, with reduced tumor regrowth after treatment, in comparison with rapamycin (14, 17, 45).

Hence, mTORC1 inhibitors have been an important therapeutic advance for a variety of tumor types, but minimal or greater residual disease is nearly always present. Consequently, MRD is particularly common with rapamycin treatment, meaning that lifelong therapy is required, with consequent long-term toxicity, including stomatitis, rash, and fatigue (46, 47).

These clinical findings motivated us to examine mechanisms of resistance to mTORC1 inhibitors in multiple tumor/cancer cell lines, as reported here. We generated 11 RP or MRD models by long-term mTORC1 inhibitor exposure either in vitro or in vivo. Four of 7 RP cell lines developed *MTOR* mutations in the FRB domain, and one also acquired a mutation in the kinase domain. Cryo-EM structural studies have shown that FKBP12-rapamycin and the mTOR signaling motif of mTORC1 substrates compete for binding to the FRB domain or Raptor (32, 48). We identified 3 RP cell lines with acquired mutations at Ser2035 and another one with mutations at Ala2034. However, 2 other sequenced RP lines showed no acquired mutations in *MTOR*, *FKBP12*, or other pathway members. We found that Ser2035-Cys and Ser2035-Phe completely abolished binding of rapamycin-FKBP12 to mTORC1 and dramatically decreased the binding of bi-steric mTORC1 inhibitor-FKBP12, whereas Ser2035-Pro had smaller effects on these binding events, as assessed in co-IP experiments (Figure 1F; Figure 2, C and D; and Supplemental Figure 2, B–D). All the RP/MRD cells, with or without *MTOR* mutations, were much less sensitive to all 3 mTORC1 inhibitors (rapamycin and 2 bi-sterics) in comparison with their parental counterpart cells. FKBP12 expression was also sharply reduced in 1 RP line (Supplemental Figure 2F). S6K and 4EBP1 had persistent phosphorylation in the RP/MRD cells without *MTOR* mutations (HCV29RP, 97-1RP), and yet the rapamycin-FKBP12 complex still bound to MTOR. These findings might reflect a conformational change in mTORC1 induced by long-term rapamycin treatment (32, 49). Further investigation is required to address this hypothesis.

As RP/MRD cells without *MTOR* mutations were also persistent to mTORC1 inhibitors, we hypothesized that epigenetic changes contributed to the RP/MRD phenotype (50, 51). Cancer cells predominantly use anaerobic glycolysis, not OXPHOS, even in the presence of abundant oxygen (52). Moreover, enhanced glycolysis was a hallmark of TSC1/TSC2-knockout cells from their earliest derivation (53). However, metabolomic analysis revealed that RP/MRD cells, with or without *MTOR* mutations, had metabolically reprogrammed from glycolysis to OXPHOS. Integrated multiomic analysis led us to discover that MYC protein expression was enhanced in all RP/MRD cell lines and was the apparent driver of upregulation of mitochondrial OXPHOS and increased ATP levels, as verified by Seahorse assay and mitochondria staining. Concurrent with MYC enhancement, SWI/SNF activity was also increased by expression analysis, in a potentially cooperative mechanism. Chromatin accessibility is regulated in different levels by different mechanisms, including chromatin remodelers (such as SWI/SNF), pioneer factors, histone modifications, and more (25–27). Hence, other mechanisms may exist in reshaping chromatin status in RP cells.

We examined whether this shift to OXPHOS induced by chronic rapamycin therapy might represent a therapeutic vulnerability. We found that RP/MRD cells were more sensitive to an oxidative phosphorylation inhibition (IACS-010759), and to an SWI/SNF inhibitor (AU-15330), than the starting lines, and were also extremely sensitive to cotreatment with a bi-steric mTORC1 inhibitor. In addition, we observed that the RP cells were strikingly sensitive to cuproptosis induction with ES and Cu, in comparison with the starting lines. Upregulated ROS and lipid peroxidation have been linked to cuproptosis. However, the relationship between ROS, lipid peroxidation, and cuproptosis requires further experimental clarification given that ROS and lipid peroxidation are highly dynamic processes within cells and can be influenced by numerous factors (34).

In summary, long-term mTORC1 inhibitor treatment with rapamycin led to an RP phenotype, with broad chromatin, expression, and metabolic rewiring in comparison with the starting mTORC1-activated cell lines. These RP cells demonstrated markedly enhanced OXPHOS in comparison with the starting lines and were exquisitely sensitive to combination treatments with a bi-steric mTORC1 inhibitor and either SWI/SNF inhibitor or mitochondrial complex I inhibitor, through induction of cuproptosis. Preliminary clinical data have been presented from the first-in-human study of the bi-steric mTORC1 inhibitor RMC-5552 (45). Hence, this therapeutic approach may be valuable in treatment of rapamycin persistence arising in TSC and in patients with cancer receiving mTORC1 inhibitors.

## Methods

### Sex as a biological variable.

Male 6-week-old C57BL/6J mice (The Jackson Laboratory, 000664) were used as previously described (16, 17). Mouse sex was not considered as a biological variable.

### Cell lines and cell culture.

All the cell lines were maintained as previously described (17). Briefly, 105K mouse renal carcinoma kidney cyst-adenocarcinoma cells, 621-101 human AML cells (54), MEFs, and mouse kidney hybrid oncocytic/chromophobe tumor cells and human BLCA lines (HCV29, RT4, and 97-1) were maintained in DMEM with 10% FBS and 100 U/mL of pen/strep. The human HCC cell line (SNU886) was maintained in RPMI-1640 with 10% FBS and 100 U/mL of pen/strep. All the cells were incubated at 37°C in 5% CO_2_. Mycoplasma was tested routinely, and only mycoplasma-negative cells were used.

### Compounds.

The rapamycin was purchased from LC Laboratory (R-5000). Sapanisertib, RMC-4627, and RMC-6272 were provided by Revolution Medicines. SWI/SNF inhibitor (AU-15330) was purchased from MedChemExpress LLC (HY-145388). OXPHOS inhibitor was purchased from Selleck Chemical LLC (IACS-010759). All compounds used in vitro were dissolved in DMSO.

### Generation of RP cell lines and MRD cells.

RP cell lines were generated by culturing the parental cells (mentioned above) in rapamycin (500 nM). Media were changed every 3 days. MRD525 and MRD639 cells were generated by injection of 105K cells into C57BL/6J mice (The Jackson Laboratory, 000664) and treated by rapamycin (3 mg/kg, every other day, IP) and RMC-6272 (8 mg/kg) for 4 weeks followed by 2 months off treatment. Recurrent tumor nodules were harvested, digested into single-cell suspension (55), and cultured in DMEM with 10% FBS and 100 U/mL of pen/strep.

### Cell survival/proliferation assay.

Cell survival and proliferation was measured by crystal violet staining as previously described (17). Cells were seeded in 96-well plates and treated with different concentrations of compounds for 3 days. Cells were then fixed with 10% formalin and stained with 0.05% crystal violet, which was then extracted and assessed by densitometry at 540 nM. The IC_50_ and growth inhibition curves were calculated using GraphPad Prism 10.0.

### Colony formation assay.

To evaluate the long-term effects of different compounds on cell proliferation, 2D clone formation assay was performed as previously described. A total of 200–1,000 cells were seeded in 10 cm plates and treated by different compounds for 14 days. Clones were fixed using 10% formalin and stained by 0.05% crystal violet. Clone numbers of size > 1 mm were counted manually and analyzed using Prism 10.0.

### Immunoblot.

Cell lysates were prepared as previously described. Primary antibodies against mTOR (2983S), Raptor (11816S), mLST8 (3274S), TSC2 (4308S), TSC1 (4906S), AKT (4685S), p-AKTS473 (4060S), S6K (2708S), p-S6K^T389^ (9234S), S6 (4858S), p-S6^S240,244^ (5364S), 4EBP1 (9644S), p-4EBP1^T37,46^ (2855S), p-4EBP1^S65^ (9451S), p-4EBP1^T70^ (9455S), β-actin (3700S), TOM20 (42406S), BRG1 (49360S), MYC (13987S), and DLAT (12362S) and anti-rabbit/mouse secondary antibodies were purchased from Cell Signaling Technology (CST). Antibody against FKBP12 (AB92459) was purchased from Abcam.

### MTOR IPs and in vivo FKBP12 competitive binding assay.

Cells were treated with rapamycin, sapanisertib, RMC-4627, or RMC-6272 for 20 minutes and then lysed in 0.3% CHAPS buffer (56). Protein (1 mg) was incubated with 5 μg anti-mTOR rabbit monoclonal antibody (CST, 2972S) or control rabbit IgG at 4°C overnight with gentle agitation. A total of 50 μL protein G agarose slurry was added, and samples were incubated for 2 hours at 4°C with gentle agitation. Immunocomplexes were pelleted by spinning down and washed using 0.3% CHAPS buffer and washing buffer 2 at 4°C (56). Then 1× Laemmli sample buffer (Bio-Rad Laboratories, 1610737) was added. Lysates were boiled and subjected to Western blot. mTOR, Rictor, Raptor, DEPTOR, PRAS40, and FKBP12 were probed.

### IHC.

IHC was performed on FFPE slides, as previously described. Primary antibodies against TOM20 (42406S, 1:400) and BRG1 (49360S, 1:200) were from CST. Anti-rabbit secondary antibody was obtained from Vector Laboratories (MP-7401-50) and detected by ImmPACT AEC Peroxidase (HRP) Substrate Kit (SK4205). Slides were then counterstained with Mayer’s hematoxylin (Agilent Technologies, S330930-2) and mounted using Fluoromount-G (SouthernBiotech, 0100-01).

### Mitochondrial staining and quantification.

Mitochondria were detected by MitoTracker staining using MitoTracker Red CMXRos (100 nM, Thermo Fisher Scientific, M7512) per the manufacturer’s recommendations. After 4% paraformaldehyde fixation, cells were then stained for F-actin using CytoPainter, Phalloidin-iFluor 488 reagent (Abcam, ab176753), and DAPI. Images were obtained using confocal microscope (Olympus Fluoview FV10i), followed by quantitative analysis of mitochondrial morphology, dynamics, and function using Mitochondria Analyzer. Mitochondria membrane potential was measured by JC-1 staining (10 μM, Abcam, ab113850) according to the manufacturer’s recommendations. Mitochondria Analyzer (57) was used for quantification of mitochondria numbers.

### Seahorse Mito Stress assay.

The same number of cells were seeded in Seahorse XF24 Cell Culture Microplates (Agilent, 100777-004) and regular 24-well plates in growth media (mentioned above) and incubated at 37°C overnight to allow the cells to adhere. Cells were seeded in Seahorse XF24 Cell Culture Microplates in growth media (mentioned above) and incubated at 37°C overnight to allow the cells to adhere. The next day, growth media were replaced by Seahorse XF Phenol Red-free DMEM, pH 7.4 (Agilent, 103575-100), supplemented with glucose (10 mM), sodium pyruvate (1 mM) and l-glutamine (2 mM). The OCR and extracellular acidification rate were measured using a Seahorse XFe24 analyzer (Agilent), with addition of oligomycin (1 μM), FCCP (1 μM), and antimycin A and rotenone (0.5 μM each). Assay settings were the following: 3 minutes mix, no wait, 3-minute measurement, repeated 3 times at baseline and after each addition. Cell numbers were counted using the cells seeded in the regular 24-well plates before Seahorse assay. OCR was then normalized to the cell numbers.

### Cellular ROS assay.

The ROS level was measured using DCFDA/H2DCFDA cellular ROS assay kit (Abcam, AB113851). Briefly, cells were seeded in a 96-well plate and treated with the compounds indicated. After treatment, cells were incubated with DCFDA 10 μM for 45 minutes at 37°C in 5% CO_2_ followed by washing. Fluorescence spectroscopy was performed with excitation/emission at 485/535 nm.

### Glycolysis/OXPHOS assay.

The metabolism shift of RP/MRD cells was detected by Glycolysis/OXPHOS Assay (DOJINDO, G270) per the manufacturer’s manual. Briefly, cells were seeded in a 96-well white microplate. The next day, media were removed, and 100 μL of 1.25 μM oligomycin solution or 100 μL of 2-DG (22.5 mM solution) was added. DMEM without glucose (Thermo Fisher Scientific, 11-966-025) with 10% FBS was used. After 5-hour incubation, 20 μL of cell culture supernatant of each well was collected and diluted 10-fold with double-distilled H_2_O. The diluted samples were added in a 96-well plate and incubated for 30 minutes at 37°C. The absorbance at 450 nm was measured. ATP working solution (100 μL) was added to the cells remaining in each well of the microplate followed by 10 minutes of incubation at 25°C. Luminescence was measured using a plate reader. The absorbance and luminescence values of with and without oligomycin and 2-DG were compared.

### Cell copper assay.

Copper ion content was measured using Cell Copper Colorimetric Assay Kit (Invitrogen, EEA007). Then 100 μL lysis buffer was added to 1 × 10^6^ cells. The cells were lysed on ice for 10 minutes followed by 10 minutes of centrifugation at 12,000*g*. The supernatant was collected. Either 100 μL of standard copper solutions or 100 μL of cell lysates was mixed with 50 μL of chromogenic solution followed by 5-minute incubation at 37°C. OD value at 580 nm was measured using microplate reader. Copper concentration was calculated based on the standard curve.

### Lipid peroxidation assay.

Lipid peroxidation assay (Abcam, 118970) was used for the measurement of lipid peroxidation. A total of 2 × 10^6^ cells for each condition were harvested and washed by PBS. Cells were lysed in 303 μL Lysis Solution (300 μL lysis buffer + 3 μL BHT stock) using a Dounce homogenizer on ice. Cell lysates were centrifuged at 12,000*g* for 10 minutes and the supernatant was collected. A total of 200 μL supernatant or standard was mixed with 600 μL of Developer VII/TBA reagent and incubated at 95°C for 60 minutes followed by 10 minutes cooling on ice. A total of 200 μL of reaction mix for each sample was transferred to 96-well plate and absorbance was measured at OD 532 nm.

### ATP measurement.

CellTiter-Glo 2.0 Cell Viability Assay (Promega, G9242) was used to measure ATP according to the manufacturer’s directions. Briefly, cells were seeded in 96-well plates and treated per directions. ATP levels were calculated based on an ATP standard curve. ATP levels were then normalized to cell number.

### Whole-exome sequencing.

Genomic DNA was extracted from cells using the QIAamp DNA Mini Kit (QIAGEN, 51304), and exome sequencing was performed by Novogene. Briefly, sequencing libraries were generated using SureSelectXT Mouse and human genome AII Exon Kit (Agilent Technologies). After purification using the AMPure XP system (Beckman Coulter) and quantification of DNA yield, libraries were sequenced on the Illumina platform. Burrows-Wheeler Aligner was utilized to map the paired-end clean reads to the mouse or human reference genome. About 45 million reads were achieved per sample. muTect was used to detect somatic single nucleotide variants. Strelka was used to detect somatic InDels.

### RNA-Seq.

Total RNA was purified using RNeasy Mini Kit (QIAGEN, 74106). RNA-Seq was performed at Novogene. A total of 1 μg RNA/sample was used as input material. Libraries were generated using NEBNext Ultra RNA Library Prep Kit for Illumina (New England Biolabs) following manufacturer’s recommendations and sequenced on Illumina NovaSeq 6000 platform. Paired-end clean reads (20 million per sample) were aligned to the reference genome hg38 or mm10 using STAR v.2.5 software. FeatureCounts was used to count the read numbers followed by reads per kilobase of exon mode per million mapped reads) calculation based on the length of each gene. DESeq2 R package (V.1.36.0) was used for differential expression analysis between 2 conditions with 2 or 3 biological replicates per condition. Genes with an adjusted *P* < 0.05 were assigned as differentially expressed. Pathway enrichment analysis (GSEA, Gene Ontology, and Kyoto Encyclopedia of Genes and Genomes) was performed.

RNA was extracted from RP cells or 105K xenograft and human TSC1^–/–^ BLCA PDX samples after tumor relapse (17).

### Multiomics.

Sequential tandem mass spectrometry analysis of polar metabolites, nonpolar lipids, proteins, and phosphorylation sites from a single sample was performed (17, 58, 59). A total of 10 million cells or 10 mg tumor tissue per sample was used. Triplicate samples were included for each condition. We added 200 μL of 1× PBS and 1.5 mL high-performance liquid chromatography–grade (HPLC-grade) methanol, followed by a vigorous vortex for 1 minute at room temperature (RT). The samples were shaken for 1 hour at RT after adding 5 mL of HPLC-grade *tert*-butyl methyl ether, anhydrous, 99.8% (Sigma-Aldrich, 34875-2L). Then 1.2 mL HPLC-grade water was added, vortexed for 1 minute, and spun for 10 minutes. The resulting upper (lipids) and middle (metabolites) phases were collected separately in 1.5 mL glass vials and dried out using SpeedVac Concentrator (Thermo Fisher Scientific, SC110A). A protein pellet at the bottom was used for both proteomics and phosphoproteomics. High-Select TiO_2_ Phosphopeptide Enrichment Kit (Thermo Fisher Scientific, PIA32993) was used to enrich phosphopeptides. The metabolite samples were resuspended in 20 μL liquid chromatography-tandem mass spectrometry–grade (LC/MS-grade) water and run as previously described. The data were analyzed using Elements for Metabolomics (Proteome Software) with the National Institute of Science and Technology (NIST) database incorporated (http://chemdata.nist.gov/mass-spc/msms-search/) followed by statistical analysis with Metaboanalyst 5.0 (http://www.metaboanalyst.ca/). The lipid samples were resuspended in 30 μL of LC/MS-grade isopropanol/methanol (1:1), and 5 μL was injected for LC-MS/MS analysis. Lipidomic data were analyzed using LipidSearch 4.1.9 software (Thermo Fisher Scientific) and Elements for Metabolomics (Proteome Software) NIST database incorporated. The protein samples were analyzed by positive ion–mode LC-MS/MS using a high-resolution hybrid QExactive HF Orbitrap Mass Spectrometer (Thermo Fisher Scientific) via higher-energy collisional dissociation with data-dependent analysis with 1 MS1 scan followed by 8 MS2 scans per cycle (top 8). MS/MS spectra were analyzed for the peptide samples with a parent ion tolerance of 18 ppm and fragment ion tolerance of 0.05 Da. Carbamidomethylation of cysteine (+57.0293 Da) was specified as a fixed modification, and oxidation of methionine (+15.9949) phosphorylation of serine/threonine/tyrosine (+79.97) as variable modifications. Results were imported into Scaffold Q+S 4.6 software (Proteome Software) with a peptide threshold of ~85% protein threshold of 95%, resulting in a peptide FDR of ~1%. Further statistical analysis was performed using Panther (http://www.pantherdb.org/) after removing contaminants such as keratins, caseins, trypsin, and BSA. Omics data were normalized to the median of each sample and then subjected to differential analysis (DESeq2) and pathway enrichment analysis.

### Prediction of potential TFs.

ChEA3 (https://maayanlab.cloud/chea3/) was used to predict the potential TFs that regulate the genes in each pathway as indicated. The top 200 predicted TFs from each pathway were used for the intersection (Venn diagram).

### CUT&RUN and data analysis.

CUT&RUN was performed using a CUTANA ChIC/CUT&RUN Kit (EpiCypher, 14-1048) per manufacturer’s protocol. One million cells were harvested for each CUT&RUN assay with duplicates for each condition. Antibodies against H3K27ac (Diagenode, C15410196) and MYC (CST, 13987S) were used. For each sample, 5 ng of the purified CUT&RUN chromatin with 0.05 ng *Escherichia coli* spike-in DNA was subjected to library preparation with a NEB Ultra II DNA Library Prep Kit, followed by sequencing (using a NovaSeq 6000 system). We obtained 20 million reads per sample (paired-end reads extending 150 bases) and aligned them to hg38 or mm10 using bowtie2.4.5 after trimming adaptor sequences (60). PCR duplicates and blacklist regions were removed from aligned data using MarkDuplicates (Picard, V.2.27.4; https://broadinstitute.github.io/picard/) and bedtools (V.2.30.0), respectively (61). Peaks were called using MACS3 (62). For visualization, Deeptools (V.3.5.0) was used to convert bam files into bigwig files (63). Downstream analysis, including heatmaps, averaged plots of signals, peak annotation, and GO pathway enrichment analysis, were analyzed using ChIPseeker (V.1.32.1), EnrichedHeatmap (V.1.26.0), and DiffBind (V.3.12.0) (64–66). ROSE was used to define enhancers and superenhancers (67). HOMER (V.4.11) was used to find motifs (68). transPlotR was used for peak visualization.

### ATAC-Seq and data analysis.

ATAC-Seq was performed using ATAC-Seq Kit (Active Motif, 53150) per manufacturer’s protocol. A total of 1 × 10^5^ cells were harvested for each assay with duplicates in each condition; 50–100 million paired-end reads were obtained for each sample. Reads were aligned to hg38 or mm10 using bowtie (V.2.4.5) after adaptor trimming by Cutadapt (V. 23.5.0). Reads aligned to mitochondria were removed using samtools (V.1.15.1). MarkDuplicates (Picard, V.2.27.4) was used to remove PCR duplicates. Peak calling was done by MACS3 after shifting the bam files (62). Peak annotation, differential peak analysis, and pathway enrichment analysis were performed using ChIPseeker (V.1.32.1), EnrichedHeatmap (V.1.26.0), and DiffBind (V.3.12.0) as mentioned above. Bam files were converted to bigwig files using Deeptools (V.3.5.0). transPlotR was used for peak visualization.

### ATAC-qPCR.

EpiQuick Chromatin Accessibility Assay Kit (EpiGenTek, P-1047) was used to assess chromatin accessibility. Briefly, 2 × 10^6^ cells of each condition were collected and lysed using 1× lysis buffer for 10 minutes on ice. After spinning down, pellets containing chromatin were washed using 1× wash buffer. The chromatin was then digested by Nse nuclease mix for 4 minutes at 37°C. RSS buffer was then added to stop the digestion, and protein was digested with proteinase K for 15 minutes at 60°C. DNA was then collected by column and subjected to qPCR analysis using the primers from the kit.

### Cuproptosis and synergy effect analysis.

Cuproptosis was explored as previously described. Cells were cultured in DMEM lacking glucose (Thermo Fisher Scientific, 11-966-025) with 10% dialyzed FBS (Thermo Fisher Scientific, 26400044), 100 μg/mL of uridine (Sigma-Aldrich, U3750), 1 mM pyruvate (Thermo Fisher Scientific, 11360070), and either 10 mM glucose (A2494001) or 10 mM galactose (Sigma-Aldrich, G5388-100G). Different concentrations of ES (MedChemExpress, HY-12040)-CuCl_2_ (Sigma-Aldrich, C3279-100G; 1:1 ratio) were added to the cells for 72 hours followed by cell viability measurement. To query the synergy effect, different concentrations of AU-15330 (MedChemExpress, HY-145388) and IACS-010759 hydrochloride (MedChemExpress, HY-112037A), with or without RMC-6272 (5 nM), were used. For rescue assay, cells were pretreated with 10 μM Ferrostatin-1 (MedChemExpress, HY-100579), 10 μM TTM (Sigma-Aldrich, 323446), or 40 μM Z-VAD-FMK (MedChemExpress, HY-16658B) overnight.

### Statistics.

Data were analyzed using GraphPad Prism 10.0. Results are presented as mean ± SD or median. All cell and Western blot experiments were repeated at least twice with separately prepared samples. For 2-group comparisons, a 2-tailed unpaired Student’s *t* test was applied. One-way ANOVA was used for multiple comparisons in experiments with more than 2 groups. *P* < 0.05 was considered statistically significant.

### Study approval.

All animal experiments were conducted using protocols approved by the Brigham and Women’s Hospital Institutional Animal Care and Use Committee.

### Data availability.

RNA-Seq data and CUT&RUN data generated in this study were uploaded into the National Center for Biotechnology Information Gene Expression Omnibus database (GSE236742, GSE270779). The cartoon of cell apoptosis was obtained from BioRender (https://www.biorender.com/). Values for all data points in graphs are reported in the Supporting Data Values file.

## Author contributions

HD conceived the project; developed experimental protocols; designed, performed, interpreted, and analyzed experiments; performed bioinformatics analyses; and wrote, edited, and reviewed the manuscript. HJL, ML, and YCY assisted with experiments. MY and JMA performed the mass spectrometry. MS and EPH discussed the project. DJK supervised the project and discussed and edited the manuscript. All authors read and approved the final manuscript.

## Funding support

- DJK and HD from Revolution Medicines.
- US Department of Defense (W81XWH-21-TSCRP-IDA, to DJK and HD).

## Supplementary Material

Supplemental data

Unedited blot and gel images

Supporting data values

## Version 1 11/24/2025

Electronic publication
