# Supplementary material for: Mitochondrial metabolic rewiring sensitizes mTORC1 inhibitor persister cells to cuproptosis
Source: JCI Insight. 2025 Nov 24;10(22):e187448. doi: 10.1172/jci.insight.187448 (PMC12643498; doi:10.1172/jci.insight.187448)
Supplement: Supplemental data [file jciinsight-10-187448-s022.pdf]

Figure S1

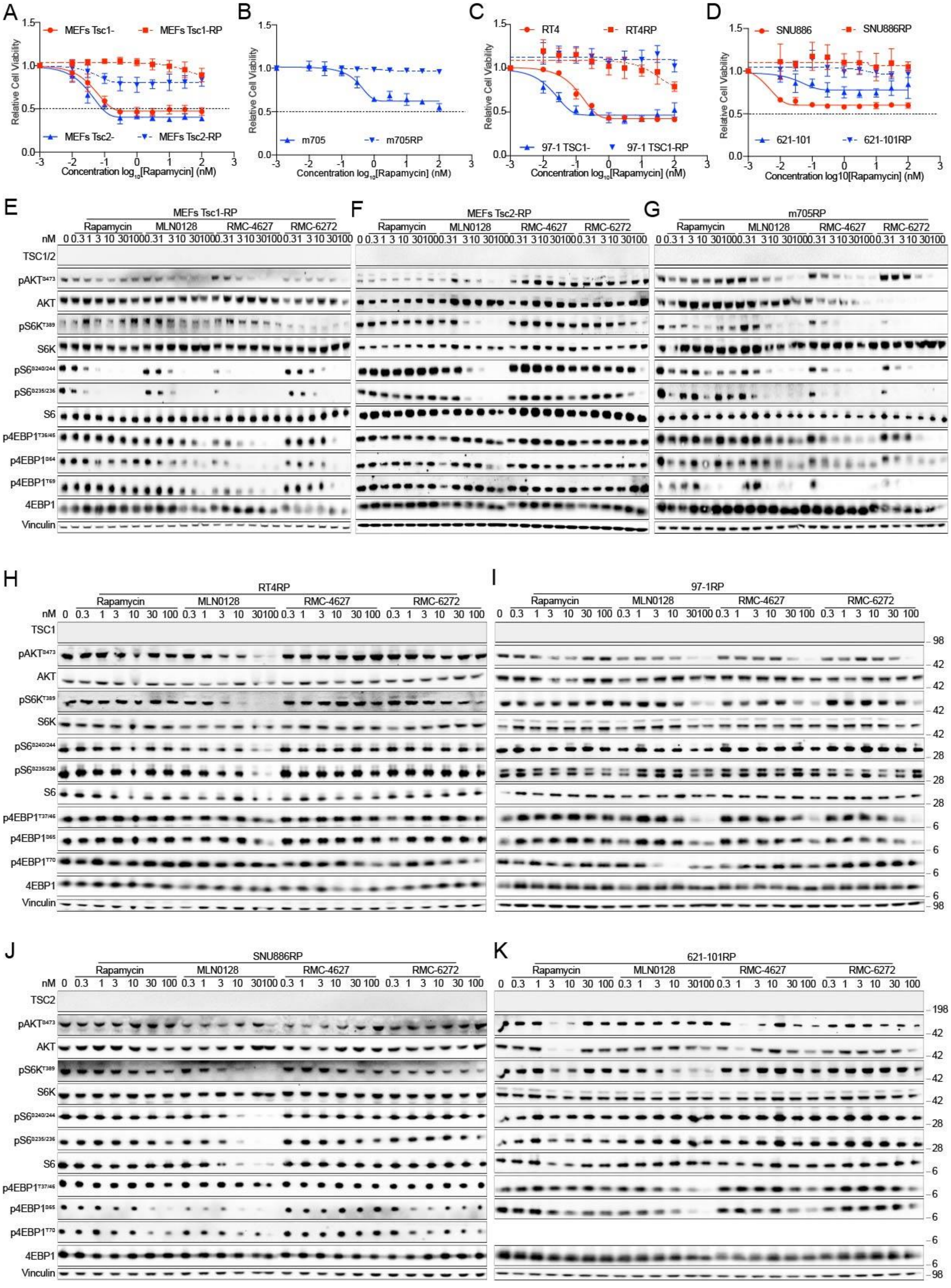
A
MEFs Tsc1-
MEFs Tsc1-RP
MEFs Tsc2-
MEFs Tsc2-RP
Relative Cell Viability
Concentration log10[Rapamycin] (nM)
B
m705
m705RP
C
RT4
RT4RP
97-1 TSC1-
97-1 TSC1-RP
D
SNU886
SNU886RP
621-101
621-101RP
Concentration log10[Rapamycin] (nM)
E
MEFs Tsc1-RP
F
MEFs Tsc2-RP
G
m705RP
Rapamycin
MLN0128
RMC-4627
RMC-6272
nM
TSC1/2
pAKT
AKT
pS6K
S6K
S6
p4EBP1
4EBP1
Vinculin
H
RT4RP
I
97-1RP
TSC1
J
SNU886RP
K
621-101RP
TSC2

Figure S1. Multiple RP models show resistance to rapamycin by IC50, and variable resistance to rapamycin and mTORC1 bi-steric inhibitors.

A - **D** IC50 of RP cell lines and their parental counterpart cells. Each dot and error bar on the curves represent mean ± SD (n = 6). **E** - **K** Immunoblot of Tsc1-RP MEFs (**E**), Tsc2-RP MEFs (**F**), m705RP (**G**), RT4RP (**H**), 97-1RP (**I**), SNU886RP (**J**), and 621-101RP (**K**) cells treated with different concentrations (nM) of mTORC1 inhibitors (4h).

Figure S2

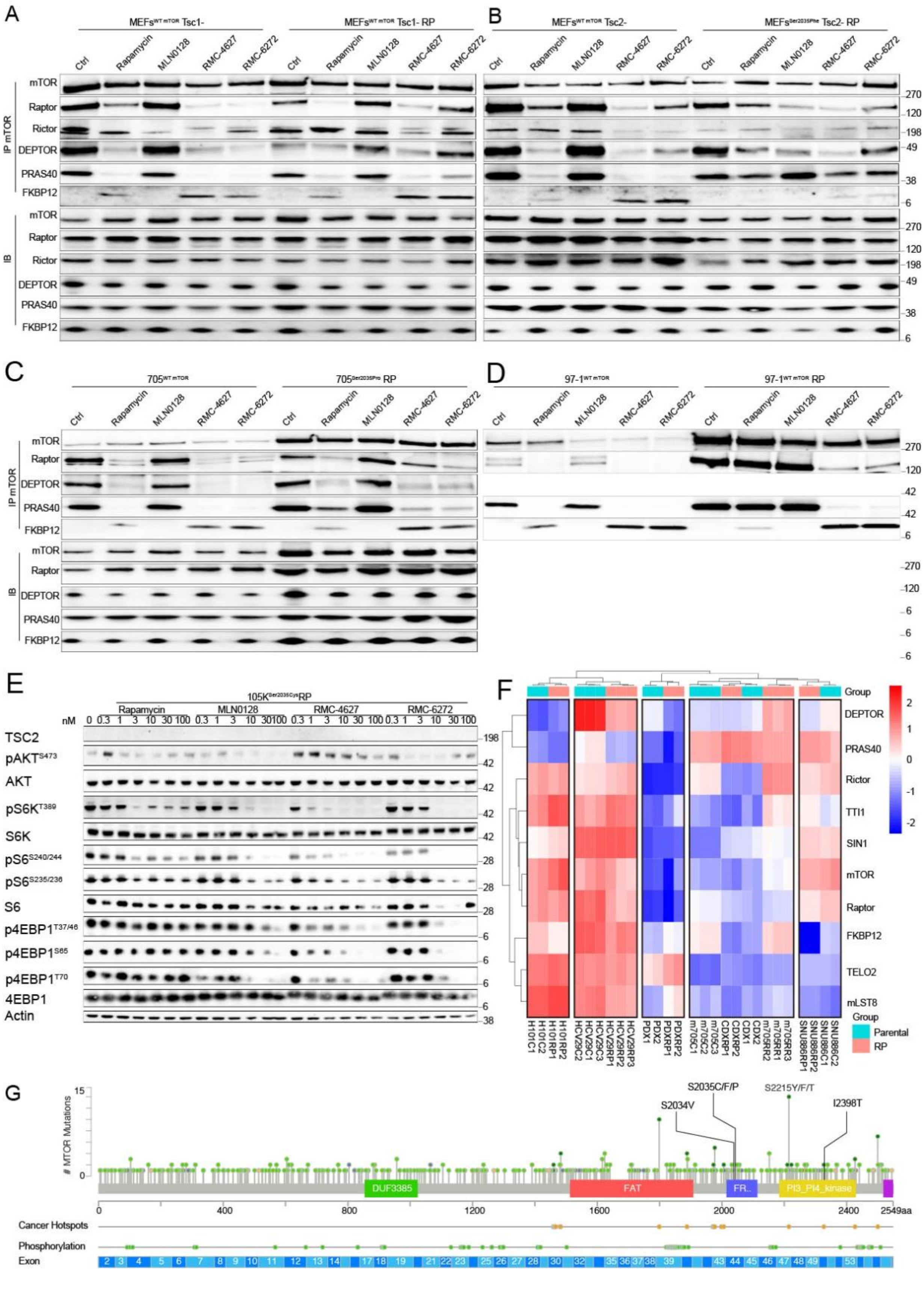
A
B
C
D
E
F
G
mTOR
Raptor
Rictor
DEPTOR
PRAS40
FKBP12
IP mTOR
IB
Ctrl
Rapamycin
MLN0128
RMC-4627
RMC-6272
TSC2
AKT
S6K
S6
4EBP1
Actin
Group
Parental
RP
SIN1
TTI1
TELO2
mLST8
S2035C/F/P
S2034V
S2215Y/F/T
I2398T
MTOR Mutations
DUF3385
FAT
PI3_PI4_kinase
Cancer Hotspots
Phosphorylation
Exon

Figure S2. RP cells with mTOR mutation have impaired Rapamycin binding affinity. **A** - **D** mTOR Co-IP of Tsc1- and Tsc1-RP MEFs (**A**), Tsc2- and Tsc2-RP MEFs (**B**), m705 and m705RP (**C**), 97-1 and 97-1RP (**D**) cells pre-treated with different mTORC1 inhibitors (10 nM, 30 mins). **E** 105KRP cells treated with different concentrations of mTORC1 inhibitors for 4h followed by immunoblot. **F** mRNA expression levels of the components of mTORC1 and mTORC2 comparing parental and RP/MRD cells assessed by RNA-Seq (n = 3 for each group). **G** Lollipop plot shows all the MTOR mutations found in all the tumors from TCGA (n = 10800). Mutations found in RP cells at different sites (S2034, S2035, S2215 and I2398) are indicated.

Figure S3

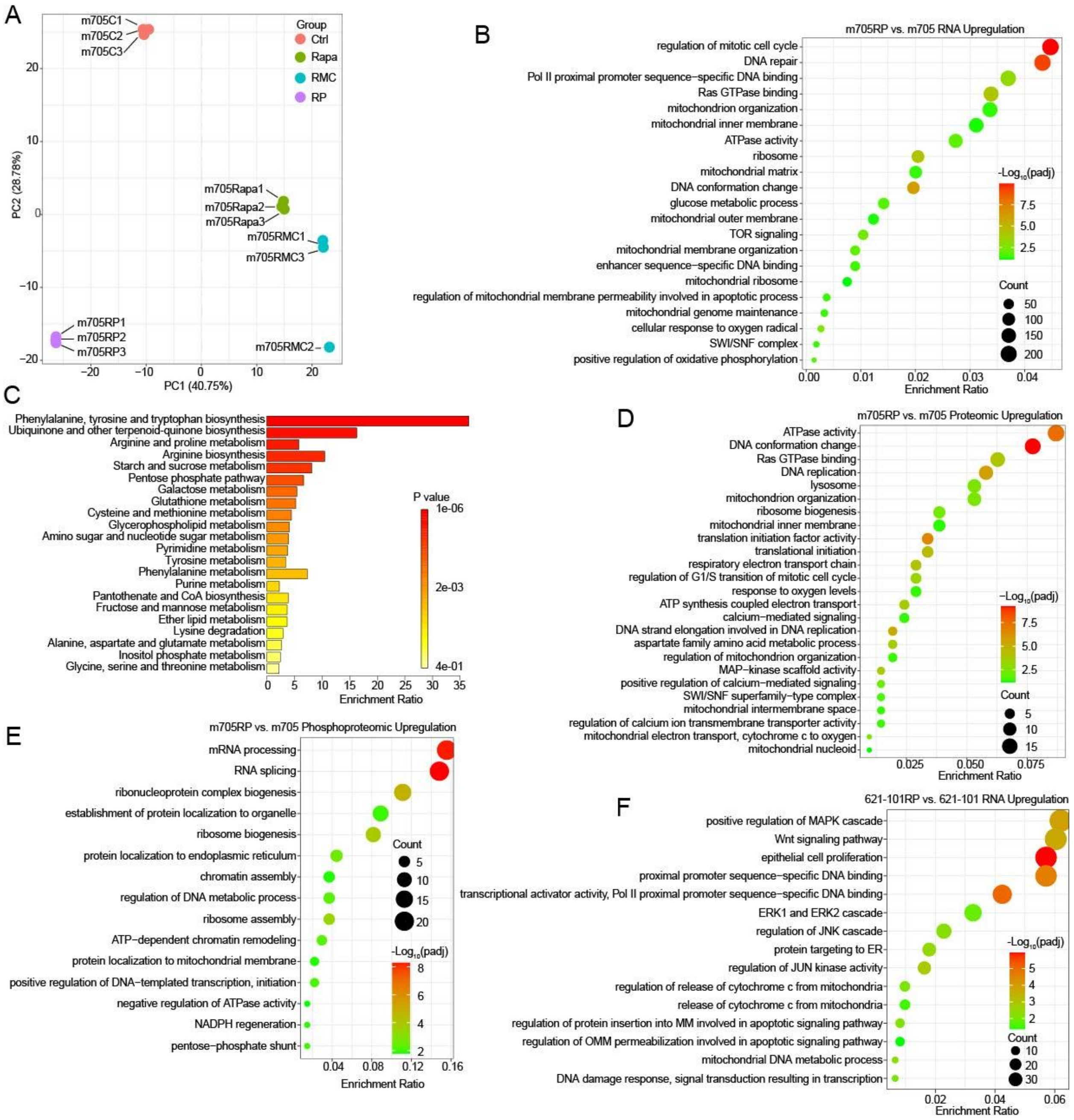

A
Group
Ctrl
Rapa
RMC
RP
m705C1
m705C2
m705C3
m705Rapa1
m705Rapa2
m705Rapa3
m705RMC1
m705RMC3
m705RMC2
m705RP1
m705RP2
m705RP3
PC1 (40.75%)
PC2 (28.78%)
B
m705RP vs. m705 RNA Upregulation
regulation of mitotic cell cycle
DNA repair
Pol II proximal promoter sequence−specific DNA binding
Ras GTPase binding
mitochondrion organization
mitochondrial inner membrane
ATPase activity
ribosome
mitochondrial matrix
DNA conformation change
glucose metabolic process
mitochondrial outer membrane
TOR signaling
mitochondrial membrane organization
enhancer sequence−specific DNA binding
mitochondrial ribosome
regulation of mitochondrial membrane permeability involved in apoptotic process
mitochondrial genome maintenance
cellular response to oxygen radical
SWI/SNF complex
positive regulation of oxidative phosphorylation
-Log10(padj)
Count
Enrichment Ratio
C
Phenylalanine, tyrosine and tryptophan biosynthesis
Ubiquinone and other terpenoid-quinone biosynthesis
Arginine and proline metabolism
Arginine biosynthesis
Starch and sucrose metabolism
Pentose phosphate pathway
Galactose metabolism
Glutathione metabolism
Cysteine and methionine metabolism
Glycerophospholipid metabolism
Amino sugar and nucleotide sugar metabolism
Pyrimidine metabolism
Tyrosine metabolism
Phenylalanine metabolism
Purine metabolism
Pantothenate and CoA biosynthesis
Fructose and mannose metabolism
Ether lipid metabolism
Lysine degradation
Alanine, aspartate and glutamate metabolism
Inositol phosphate metabolism
Glycine, serine and threonine metabolism
P value
1e-06
2e-03
4e-01
Enrichment Ratio
D
m705RP vs. m705 Proteomic Upregulation
ATPase activity
DNA conformation change
Ras GTPase binding
DNA replication
lysosome
mitochondrion organization
ribosome biogenesis
mitochondrial inner membrane
translation initiation factor activity
translational initiation
respiratory electron transport chain
regulation of G1/S transition of mitotic cell cycle
response to oxygen levels
ATP synthesis coupled electron transport
calcium−mediated signaling
DNA strand elongation involved in DNA replication
aspartate family amino acid metabolic process
regulation of mitochondrion organization
MAP−kinase scaffold activity
positive regulation of calcium−mediated signaling
SWI/SNF superfamily−type complex
mitochondrial intermembrane space
regulation of calcium ion transmembrane transporter activity
mitochondrial electron transport, cytochrome c to oxygen
mitochondrial nucleoid
-Log10(padj)
Count
Enrichment Ratio
E
m705RP vs. m705 Phosphoproteomic Upregulation
mRNA processing
RNA splicing
ribonucleoprotein complex biogenesis
establishment of protein localization to organelle
ribosome biogenesis
protein localization to endoplasmic reticulum
chromatin assembly
regulation of DNA metabolic process
ribosome assembly
ATP−dependent chromatin remodeling
protein localization to mitochondrial membrane
positive regulation of DNA−templated transcription, initiation
negative regulation of ATPase activity
NADPH regeneration
pentose−phosphate shunt
Count
-Log10(padj)
Enrichment Ratio
F
621-101RP vs. 621-101 RNA Upregulation
positive regulation of MAPK cascade
Wnt signaling pathway
epithelial cell proliferation
proximal promoter sequence−specific DNA binding
transcriptional activator activity, Pol II proximal promoter sequence−specific DNA binding
ERK1 and ERK2 cascade
regulation of JNK cascade
protein targeting to ER
regulation of JUN kinase activity
regulation of release of cytochrome c from mitochondria
release of cytochrome c from mitochondria
regulation of protein insertion into MM involved in apoptotic signaling pathway
regulation of OMM permeabilization involved in apoptotic signaling pathway
mitochondrial DNA metabolic process
DNA damage response, signal transduction resulting in transcription
-Log10(padj)
Count
Enrichment Ratio

Figure S3. RP/MRD cells have distinct profiling in comparison to parental or short-term Rapamycin treated cells.

A PCA plot of RNA-Seq data for m705RP cells and m705 treated by short-term mTORC1 inhibitors (10 nM, 24h). **B** - **E** Pathway enrichment analysis of RNA-seq (**B**), metabolomic (**C**), proteomic (**D**) and phosphoproteomic (**E**) data comparing m705RP and m705 cells (n = 3 for each group). **F** GSEA for RNA-Seq data of H101RP and H101 (n = 2 for each group).

Supplemental Figure 4

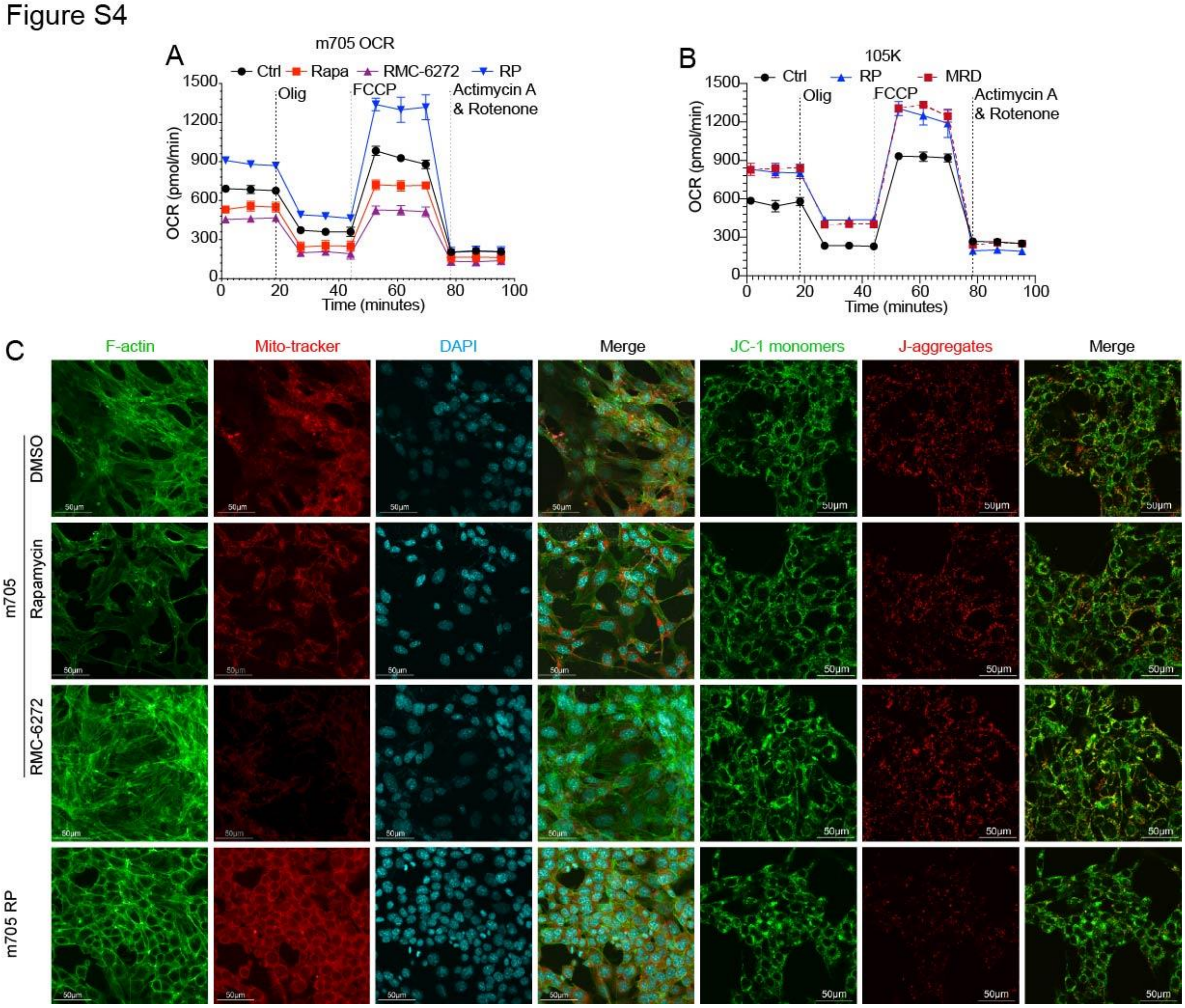

Figure S4. RP/MRD cells have increased mitochondrial function.

A, **B** OCR measurement of m705RP (**A**), 105KRP, MRD525 cells (**B**) and their parental counterpart cells with or without short-term mTORC1 inhibitors (10 nM, 24h). **C** Mito-tracker and JC-1 staining of m705RP and m705 with or without short-term mTORC1 inhibitor treatment (10 nM, 24h). Each dot and error bar on the curves represent mean ± S.D. (n =3 or n = 4). One-way ANOVA was used. * $p < 0.05$, ** $p < 0.01$, *** $p < 0.001$, **** $p < 0.0001$.

Figure S5

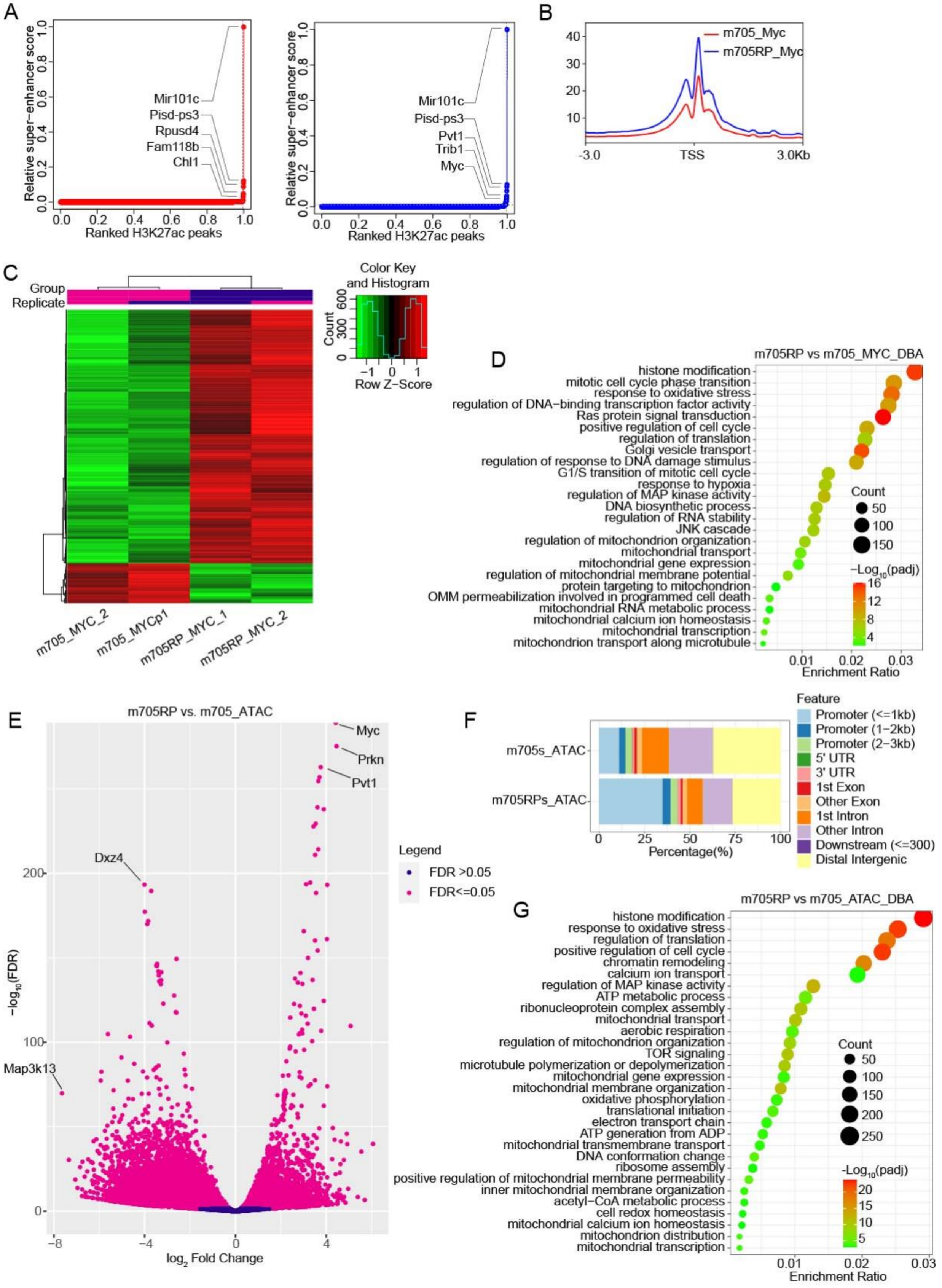

A
Relative super-enhancer score
Ranked H3K27ac peaks
Mir101c
Pisd-ps3
Rpusd4
Fam118b
Chl1
Pvt1
Trib1
Myc
B
m705_Myc
m705RP_Myc
-3.0
TSS
3.0Kb
C
Group
Replicate
Color Key and Histogram
Count
Row Z-Score
m705_MYC_2
m705_MYCp1
m705RP_MYC_1
m705RP_MYC_2
D
m705RP vs m705_MYC_DBA
histone modification
mitotic cell cycle phase transition
response to oxidative stress
regulation of DNA-binding transcription factor activity
Ras protein signal transduction
positive regulation of cell cycle
regulation of translation
Golgi vesicle transport
regulation of response to DNA damage stimulus
G1/S transition of mitotic cell cycle
response to hypoxia
regulation of MAP kinase activity
DNA biosynthetic process
regulation of RNA stability
JNK cascade
regulation of mitochondrion organization
mitochondrial transport
mitochondrial gene expression
regulation of mitochondrial membrane potential
protein targeting to mitochondrion
OMM permeabilization involved in programmed cell death
mitochondrial RNA metabolic process
mitochondrial calcium ion homeostasis
mitochondrial transcription
mitochondrion transport along microtubule
Count
-Log10(padj)
Enrichment Ratio
E
m705RP vs. m705_ATAC
Myc
Prkn
Pvt1
Dxz4
Map3k13
Legend
FDR >0.05
FDR<=0.05
-log10(FDR)
log2 Fold Change
F
m705s_ATAC
m705RPs_ATAC
Percentage(%)
Feature
Promoter (<=1kb)
Promoter (1-2kb)
Promoter (2-3kb)
5' UTR
3' UTR
1st Exon
Other Exon
1st Intron
Other Intron
Downstream (<=300)
Distal Intergenic
G
m705RP vs m705_ATAC_DBA
histone modification
response to oxidative stress
regulation of translation
positive regulation of cell cycle
chromatin remodeling
calcium ion transport
regulation of MAP kinase activity
ATP metabolic process
ribonucleoprotein complex assembly
mitochondrial transport
aerobic respiration
regulation of mitochondrion organization
TOR signaling
microtubule polymerization or depolymerization
mitochondrial gene expression
mitochondrial membrane organization
oxidative phosphorylation
translational initiation
electron transport chain
ATP generation from ADP
mitochondrial transmembrane transport
DNA conformation change
ribosome assembly
positive regulation of mitochondrial membrane permeability
inner mitochondrial membrane organization
acetyl-CoA metabolic process
cell redox homeostasis
mitochondrial calcium ion homeostasis
mitochondrion distribution
mitochondrial transcription
Count
-Log10(padj)
Enrichment Ratio

Figure S5. Long-term rapamycin treatment induces epigenetic rewiring in RP/MRD cells.

A Super-enhancer (SE) and enhancer maps comparing m705RP (blue, right) vs m705 (red, left) cells. **B** Profiling plot of MYC CUT&RUN. **C** Heatmap shows the differential peaks. **D** Pathway enrichment analysis using the differential peaks obtained from (**C**). **E** Volcano plot shows genes with different accessibilities from ATAC-seq. **F** Distribution of ATAC-seq peaks shown in (**E**). **G** GO analysis using the differential peaks obtained from ATAC-seq.

Figure S6

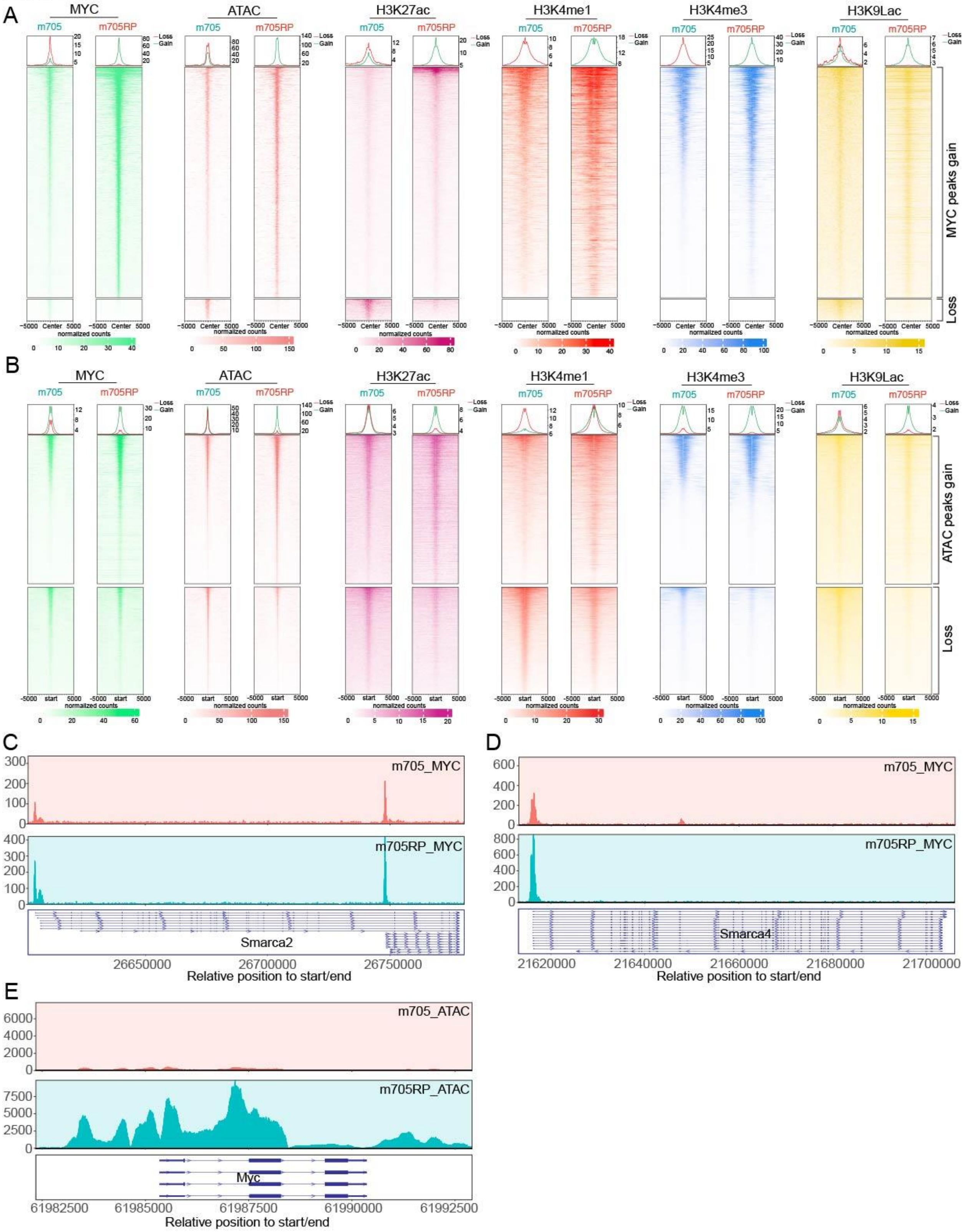
A
MYC
ATAC
H3K27ac
H3K4me1
H3K4me3
H3K9Lac
m705
m705RP
Loss
Gain
MYC peaks gain
Loss
normalized counts
B
MYC
ATAC
H3K27ac
H3K4me1
H3K4me3
H3K9Lac
ATAC peaks gain
Loss
C
m705_MYC
m705RP_MYC
Smarca2
Relative position to start/end
D
m705_MYC
m705RP_MYC
Smarca4
Relative position to start/end
E
m705_ATAC
m705RP_ATAC
Myc
Relative position to start/end

Figure S6. Cooperation of SWI/SNF and MYC re-shapes chromatin landscapes.

A Profiling heatmap of RNA-seq, MYC, H3K27ac, H3K4me1, H3K4me3, H3K9Lac CUT&RUN and ATAC-seq, aligned to the differentially expressed MYC CUT&RUN peaks. **B** Profiling heatmap of RNA-seq, MYC, H3K27ac, H3K4me1, H3K4me3, H3K9Lac CUT&RUN and ATAC-seq, aligned to the differentially expressed ATAC-Seq peaks. **C**, **D** Myc CUT&RUN signals for Smarca2 (**C**) and Smarca4 (**D**) genes. **E** ATAC-seq peaks for Myc in m705RP compared to m705 cells.

Figure S7

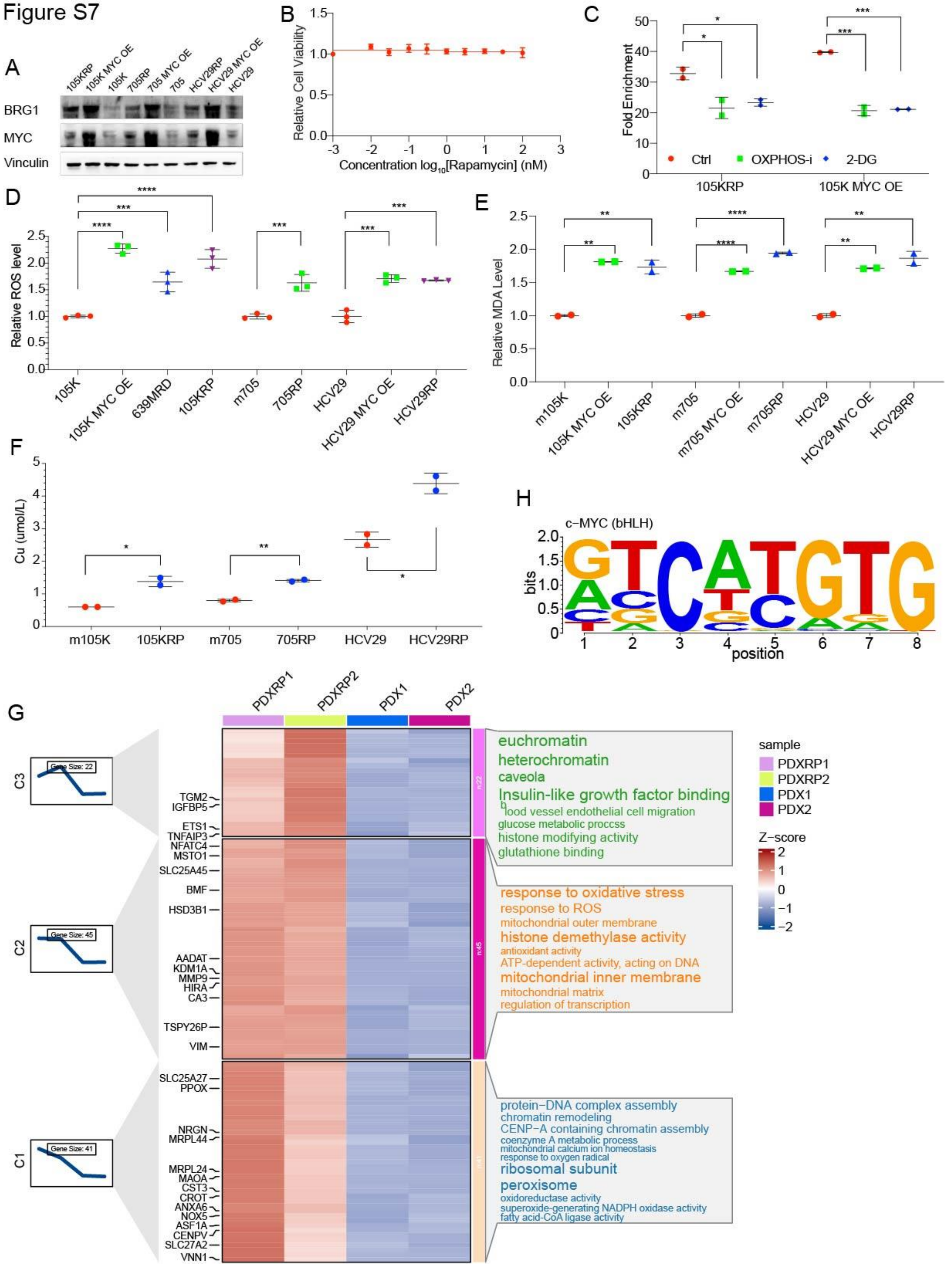
A
105KRP
105K MYC OE
105K
705RP
705 MYC OE
705
HCV29RP
HCV29 MYC OE
HCV29
BRG1
MYC
Vinculin
B
Relative Cell Viability
Concentration log10[Rapamycin] (nM)
C
Fold Enrichment
Ctrl
OXPHOS-i
2-DG
105KRP
105K MYC OE
D
Relative ROS level
105K
105K MYC OE
639MRD
105KRP
m705
705RP
HCV29
HCV29 MYC OE
HCV29RP
E
Relative MDA Level
m105K
105K MYC OE
105KRP
m705
m705 MYC OE
m705RP
HCV29
HCV29 MYC OE
HCV29RP
F
Cu (umol/L)
m105K
105KRP
m705
705RP
HCV29
HCV29RP
H
c−MYC (bHLH)
bits
position
G
PDXRP1
PDXRP2
PDX1
PDX2
C3
Gene Size: 22
C2
Gene Size: 45
C1
Gene Size: 41
TGM2
IGFBP5
ETS1
TNFAIP3
NFATC4
MSTO1
SLC25A45
BMF
HSD3B1
AADAT
KDM1A
MMP9
HIRA
CA3
TSPY26P
VIM
SLC25A27
PPOX
NRGN
MRPL44
MRPL24
MAOA
CST3
CROT
ANXA6
NOX5
ASF1A
CENPV
SLC27A2
VNN1
euchromatin
heterochromatin
caveola
Insulin-like growth factor binding
blood vessel endothelial cell migration
glucose metabolic proccss
histone modifying activity
glutathione binding
response to oxidative stress
response to ROS
mitochondrial outer membrane
histone demethylase activity
antioxidant activity
ATP-dependent activity, acting on DNA
mitochondrial inner membrane
mitochondrial matrix
regulation of transcription
protein−DNA complex assembly
chromatin remodeling
CENP−A containing chromatin assembly
coenzyme A metabolic process
mitochondrial calcium ion homeostasis
response to oxygen radical
ribosomal subunit
peroxisome
oxidoreductase activity
superoxide-generating NADPH oxidase activity
fatty acid-CoA ligase activity
sample
PDXRP1
PDXRP2
PDX1
PDX2
Z−score

Figure S7. MYC is the key driver of rapamycin persistent phenotype both in vitro and in vivo.

A Immunoblot of MYC OE in m105K, m705, and HCV29 cells. **B** IC50 of m105K MYC OE cells. **C** ATAC-qPCR of m105KRP and m105K MYC OE cells (n = 2). Fold enrichment (FE) is shown in comparison to undigested DNA from the same cells. **D-F** Measurement of ROS (n = 3), lipid peroxidation (Malondialdehyde, MDA) (n = 2) and Cu concentration (n = 2) in parental, MYC OE and RP cell lines. Each dot and error bar on the curves represent mean ± S.D. One-way ANOVA (comparing 3 or more groups) or t-test (comparing 2 groups) was used. * $p < 0.05$, ** $p < 0.01$, *** $p < 0.001$, **** $p < 0.0001$. **G** Differential gene expression and pathway enrichment analysis comparing PDX_MRD vs. PDX. **H** Motif analysis of the promoter regions of the genes from G. More details needed.

Figure S8

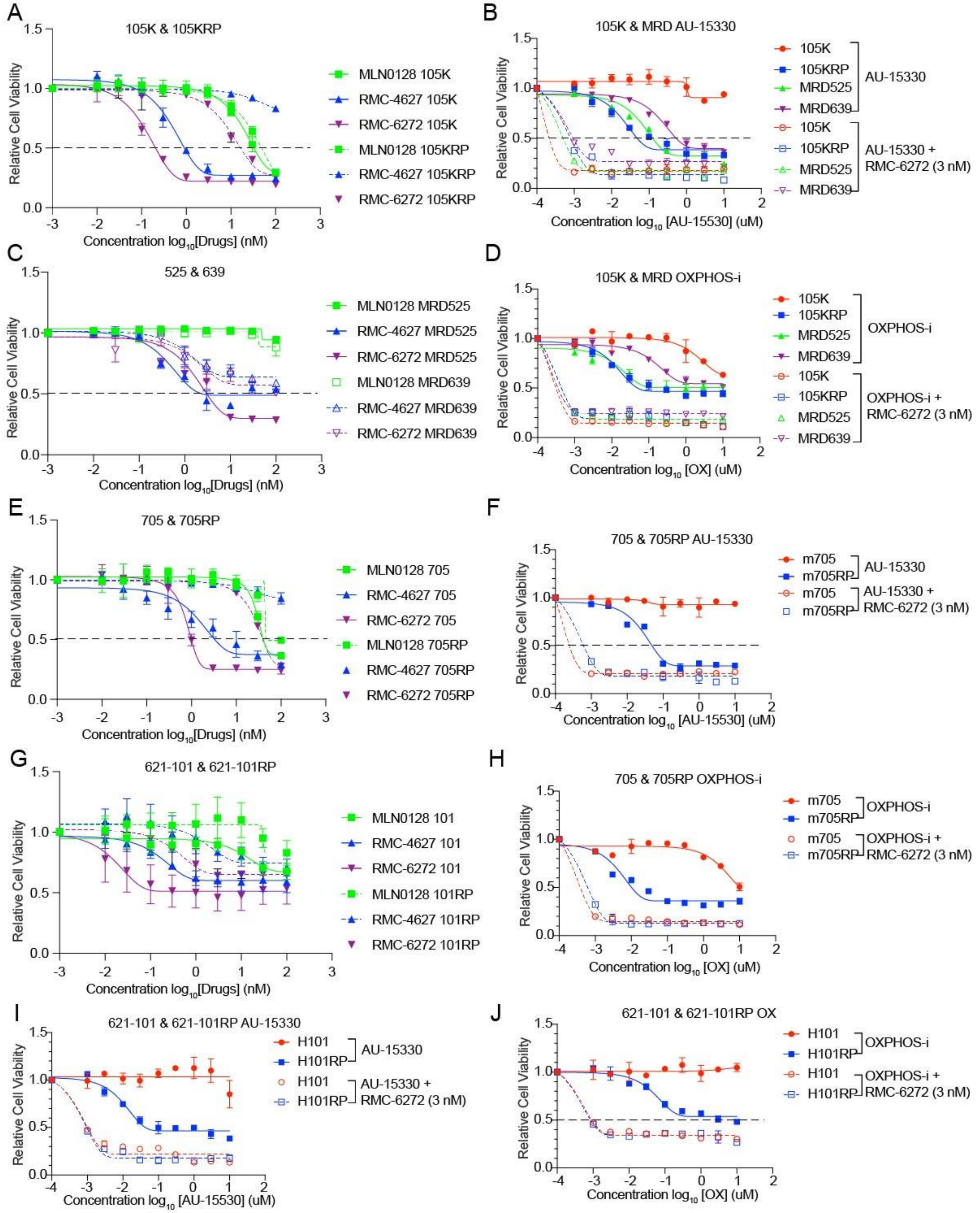
A
105K & 105KRP
Relative Cell Viability
Concentration log10[Drugs] (nM)
MLN0128 105K
RMC-4627 105K
RMC-6272 105K
MLN0128 105KRP
RMC-4627 105KRP
RMC-6272 105KRP
B
105K & MRD AU-15330
Relative Cell Viability
Concentration log10 [AU-15530] (uM)
105K
105KRP
MRD525
MRD639
AU-15330
AU-15330 + RMC-6272 (3 nM)
C
525 & 639
Relative Cell Viability
Concentration log10[Drugs] (nM)
MLN0128 MRD525
RMC-4627 MRD525
RMC-6272 MRD525
MLN0128 MRD639
RMC-4627 MRD639
RMC-6272 MRD639
D
105K & MRD OXPHOS-i
Relative Cell Viability
Concentration log10 [OX] (uM)
OXPHOS-i
OXPHOS-i + RMC-6272 (3 nM)
E
705 & 705RP
Relative Cell Viability
Concentration log10[Drugs] (nM)
MLN0128 705
RMC-4627 705
RMC-6272 705
MLN0128 705RP
RMC-4627 705RP
RMC-6272 705RP
F
705 & 705RP AU-15330
Relative Cell Viability
Concentration log10 [AU-15530] (uM)
m705
m705RP
AU-15330
AU-15330 + RMC-6272 (3 nM)
G
621-101 & 621-101RP
Relative Cell Viability
Concentration log10[Drugs] (nM)
MLN0128 101
RMC-4627 101
RMC-6272 101
MLN0128 101RP
RMC-4627 101RP
RMC-6272 101RP
H
705 & 705RP OXPHOS-i
Relative Cell Viability
Concentration log10 [OX] (uM)
OXPHOS-i
OXPHOS-i + RMC-6272 (3 nM)
I
621-101 & 621-101RP AU-15330
Relative Cell Viability
Concentration log10 [AU-15530] (uM)
H101
H101RP
AU-15330
AU-15330 + RMC-6272 (3 nM)
J
621-101 & 621-101RP OX
Relative Cell Viability
Concentration log10 [OX] (uM)
OXPHOS-i
OXPHOS-i + RMC-6272 (3 nM)

Figure S8. SWI/SNF complex inhibitor AU-15330 and OXPHOS inhibitor synergize with bi-steric mTORC1 inhibitors to suppress cell growth in RP and MRD cell lines. **A** - **J** IC50 curves of 105K, 705, and 621-101 starting and RP/MRD cell lines treated with different inhibitors. Each dot and error bar on the curves represent mean ± SD (n = 4).

Figure S9

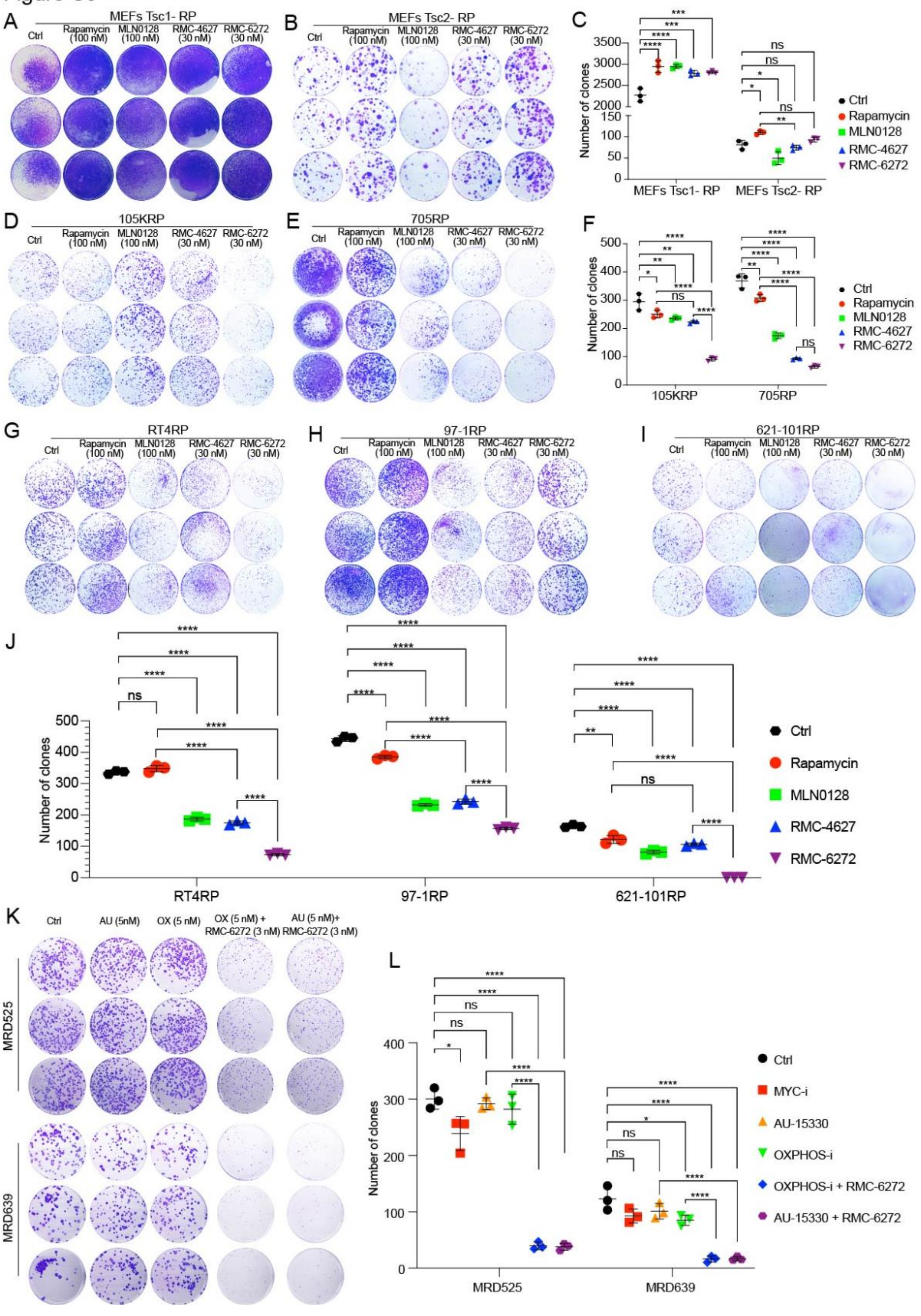

A
MEFs Tsc1- RP
Ctrl
Rapamycin (100 nM)
MLN0128 (100 nM)
RMC-4627 (30 nM)
RMC-6272 (30 nM)
B
MEFs Tsc2- RP
C
Number of clones
MEFs Tsc1- RP
MEFs Tsc2- RP
Ctrl
Rapamycin
MLN0128
RMC-4627
RMC-6272
D
105KRP
E
705RP
F
105KRP
705RP
G
RT4RP
H
97-1RP
I
621-101RP
J
RT4RP
97-1RP
621-101RP
K
AU (5nM)
OX (5 nM)
OX (5 nM) + RMC-6272 (3 nM)
AU (5 nM)+ RMC-6272 (3 nM)
MRD525
MRD639
L
MYC-i
AU-15330
OXPHOS-i
OXPHOS-i + RMC-6272
AU-15330 + RMC-6272

Figure S9. SWI/SNF complex inhibitor AU-15330 and OXPHOS inhibitor synergize with bi-steric mTORC1 inhibitors.

A - **L** RP/MRD cell lines treated with inhibitors of mTOR (Rapamycin, Sapanisertib, RMC-4627 or RMC-6272), SWI/SNF (AU-15330), mitochondria inhibitors (OXPHOS-i) or combo, as shown by 14-day low-dilution colony formation assay and quantification. Each dot and error bar on the curves represent mean ± SD ($n = 3$). One-way ANOVA was used. *$P < 0.05$, **$P < 0.01$, ***$P < 0.001$, ****$P < 0.0001$.

Figure S10

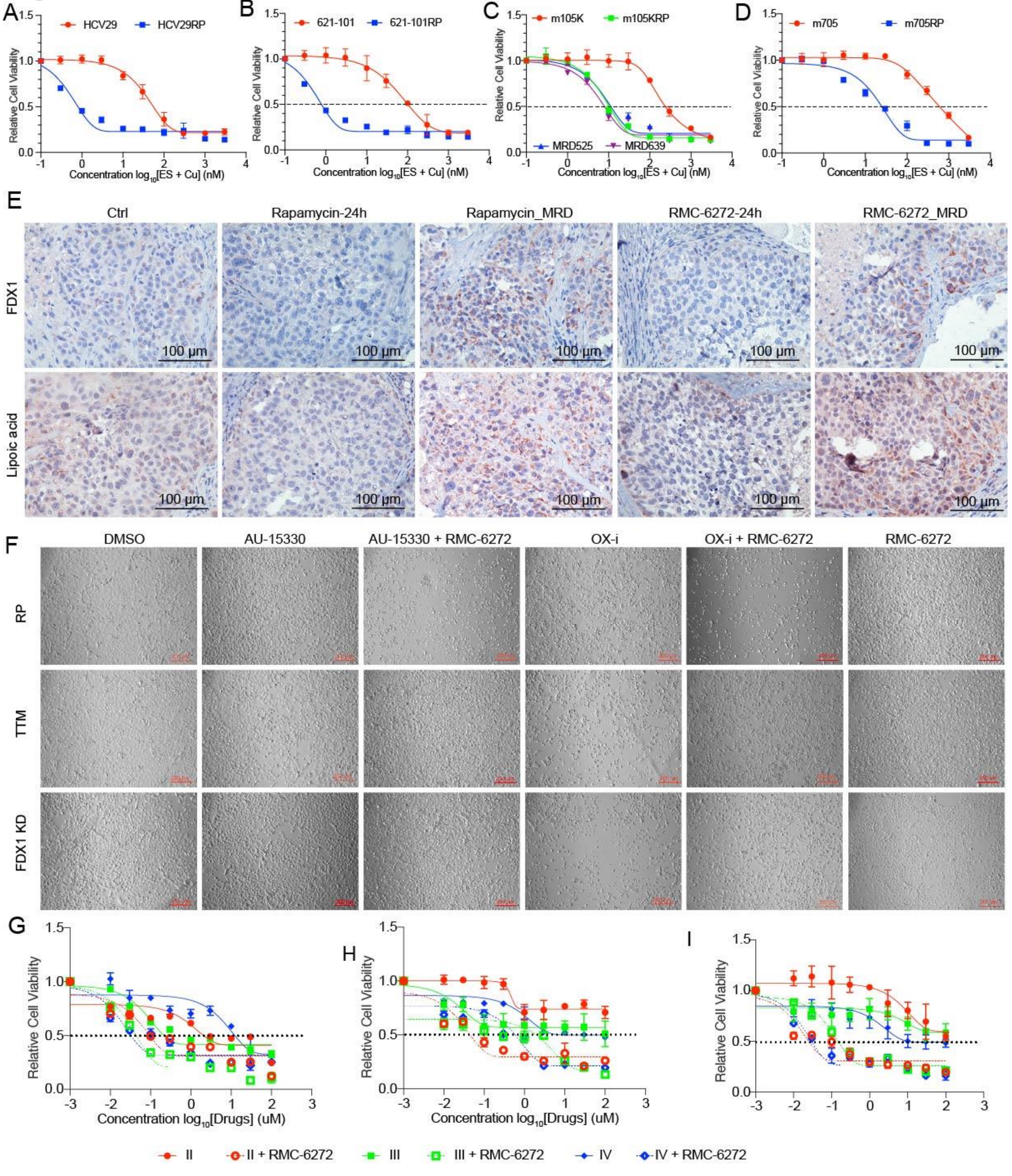
A
HCV29
HCV29RP
Relative Cell Viability
Concentration log10[ES + Cu] (nM)
B
621-101
621-101RP
C
m105K
m105KRP
MRD525
MRD639
D
m705
m705RP
E
Ctrl
Rapamycin-24h
Rapamycin_MRD
RMC-6272-24h
RMC-6272_MRD
FDX1
Lipoic acid
100 μm
F
DMSO
AU-15330
AU-15330 + RMC-6272
OX-i
OX-i + RMC-6272
RMC-6272
RP
TTM
FDX1 KD
G
H
I
Concentration log10[Drugs] (uM)
II
II + RMC-6272
III
III + RMC-6272
IV
IV + RMC-6272

Figure S10. RP/MRD cells are sensitive to cuproptosis.

A, B Dose-dependent cell growth inhibition curves of HCV29RP and 621-101RP cells and their parental cell lines treated with cuproptosis inducer (ES + Cu, 1:1 ratio). **C**, **D** Cell proliferation curves of m105K, m105KRP, MRD525, MRD639, m705 and m705RP cells when treated with cuproptosis inducer. Each dot and error bar on the curves represent mean ± SD (n = 4). **E** IHC staining of cuproptosis markers FDX1 and lipid acid in BLCA tumors after short-term rapamycin or RMC-6272 treatment and in recurrent bladder cancer TSC!-mutant PDX tumors? after rapamycin or RMC-6272 treatment cessation. **F** Phase contrast photos of HCV29RP cells or HCV29RP cells with FDX1 KD treated as indicated. **G-I** IC50 curves of 105KRP, MRD639, and 105K MYC OE cells treated with mitochondrial complex II, III, IV inhibitors with or without RMC-6272 (n = 3).

Supplementary Table 1. Summary of RP/MRD cells.

Cell line	Genotype & cell type	Treatment	mTOR Mutation	AA Change	IC50 of Rapamycin		IC50 of Sapanisertib		IC50 of RMC-4627		IC50 of RMC-6272	
					Parental	RP	Parental	RP	Parental	RP	Parental	RP
Tsc1-/- MEFs	Tsc1- mouse fibroblasts	Rapamycin 500 nM, > 3 months	Not* found	—	0.3	—	3	—	0.3	—	0.08	—
Tsc2-/- MEFs	Tsc2- mouse fibroblasts		C6104T*	Ser2035Phe	0.1	—	3	10	0.05	—	0.03	—
105K Tsc2-	Tsc2- mouse kidney tumors		C6104G*	Ser2035Cys	0.5	—	30	50	1	—	0.3	1
705 Tsc2-	Tsc2- mouse kidney tumors		T6103C*	Ser2035Pro	—	—	50	50	3	—	1	10
HCV29 TSC1-	Tsc1- human BLCA		Not found*	—	—	—	30	—	0.05	—	0.03	0.3
RT4 TSC1-	Tsc1- human BLCA		C6101T* T7193C*	A2034V I2398T	0.5	—	80	100	1.5	—	0.8	8
97-1 TSC1-	Tsc1- human BLCA		Not found*	—	—	—	30	—	0.3	—	0.8	—

621-101 TSC2	Tsc2- human AML		Not sequenced	—	—	—	—	—	—	—	—	—
SNU-886 TSC2-	TSC2- human HCC		Not sequenced	—	—	—	—	30	1	—	0.5	—
MRD525	MRD of 150K CDX	3mg/kg, 1m	Not sequenced	—	0.5	—	30	—	1	10	0.3	3
MRD639	MRD of 150K CDX	8mg/kg,1m	Not sequenced	—	—	—	—	—	—	—	—	—

—: no IC50 can be reached

*: Whole exon sequencing was performed

#this sample also had marked reduction in expression of FKBP12
