# Supplementary material for: Mitochondrial metabolic rewiring sensitizes mTORC1 inhibitor persister cells to cuproptosis
Source: JCI Insight. 2025 Nov 24;10(22):e187448. doi: 10.1172/jci.insight.187448 (PMC12643498; doi:10.1172/jci.insight.187448)

Figure 1E. HCV29RP$^{wt\ mTOR}$ Diff concentrations

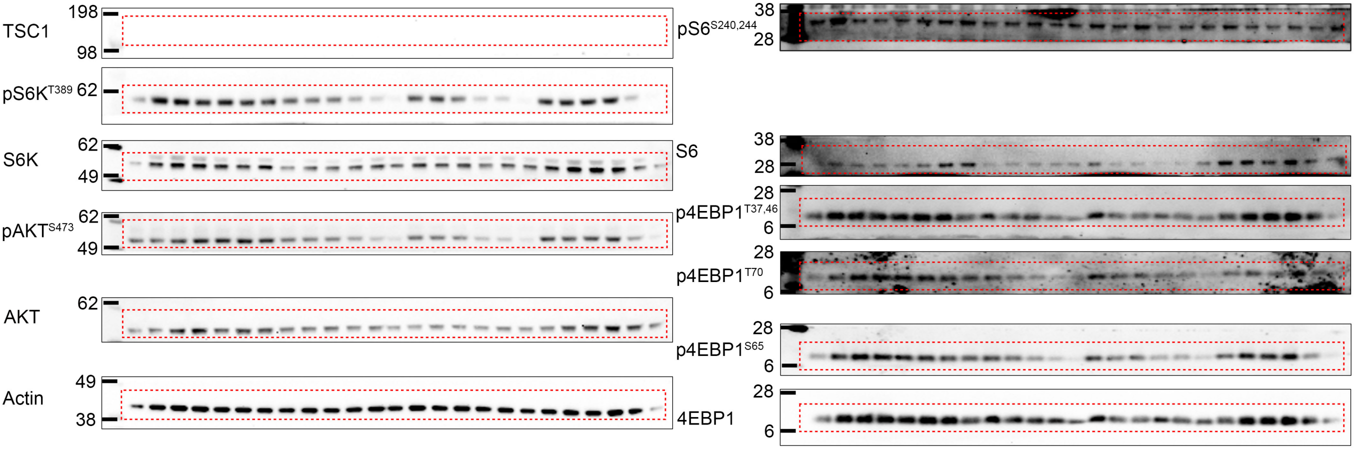

Figure 2A MRD525 Diff Concentrations

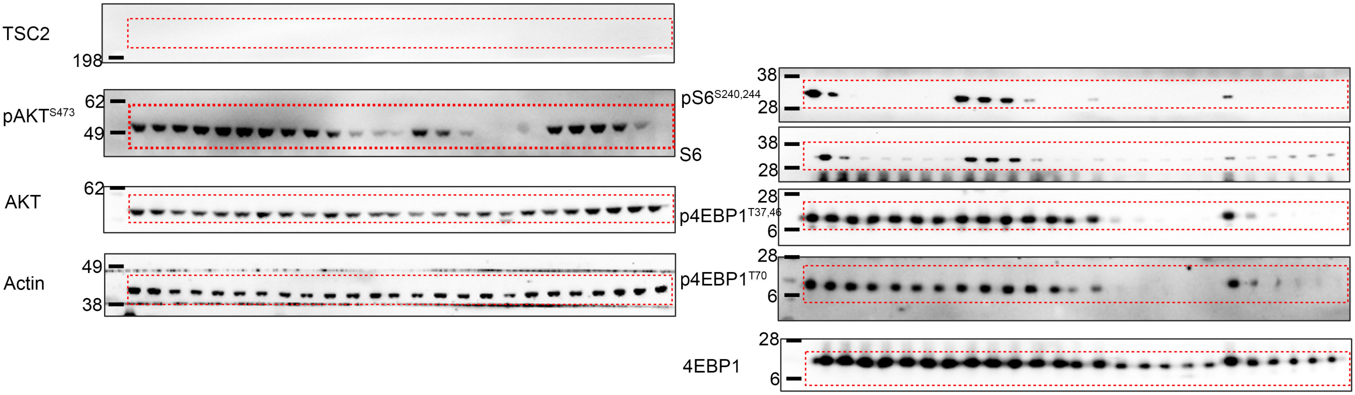

Figure 2B MRD639 Diff cons.

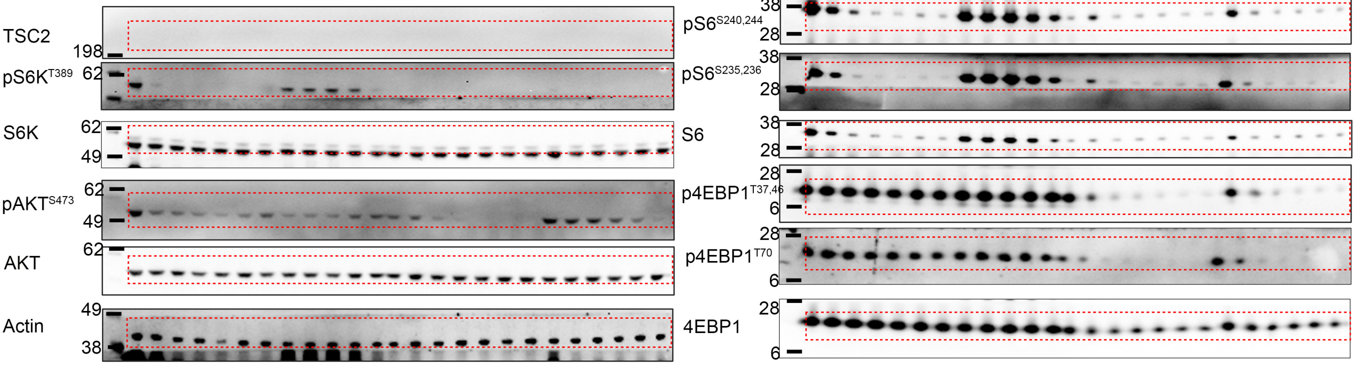

Figure 1F. HCV29RP mTOR IP

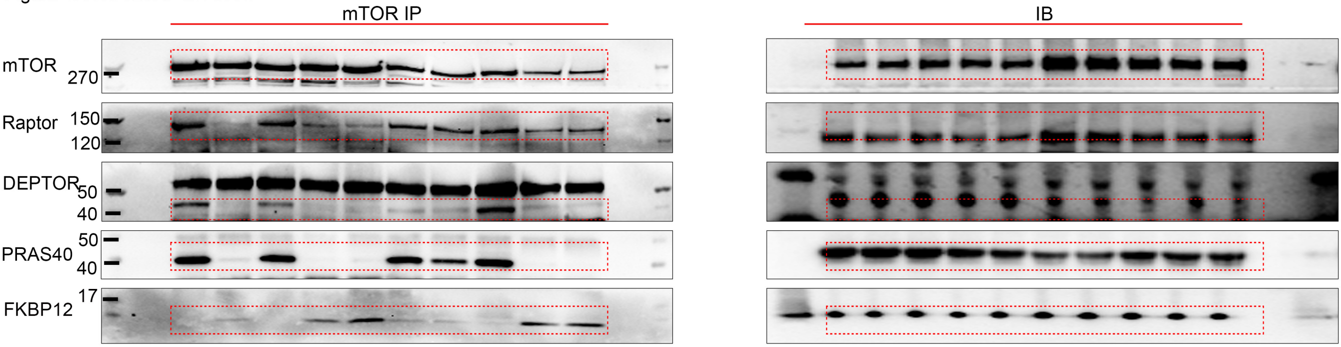

Figure 2D. 105KRP mTOR IP

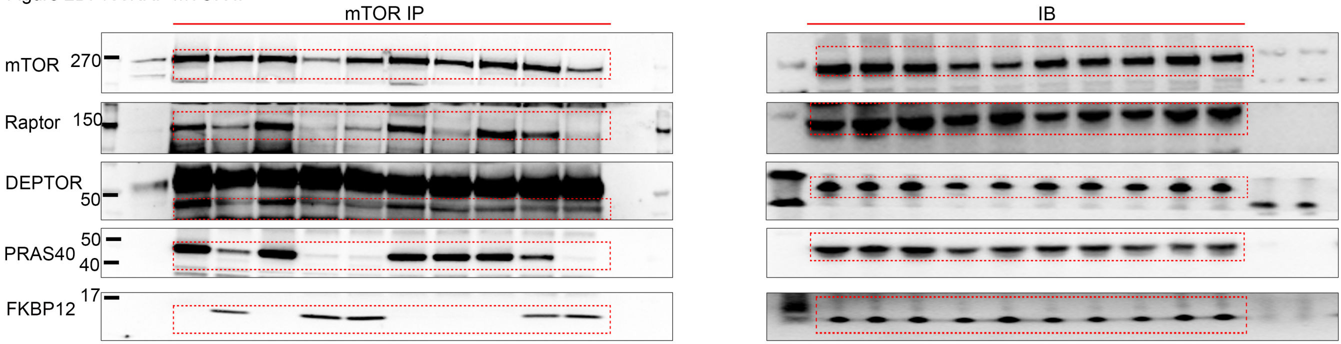

Figure 2C. MRD mTOR IP

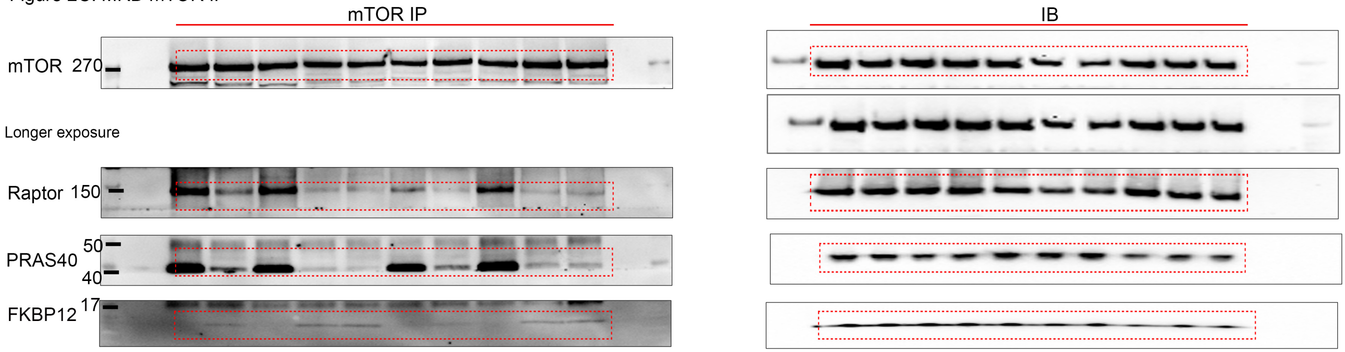

Figure S1E. TSC1 MEFs RP$^{wt\ mTOR}$ Diff concentrations

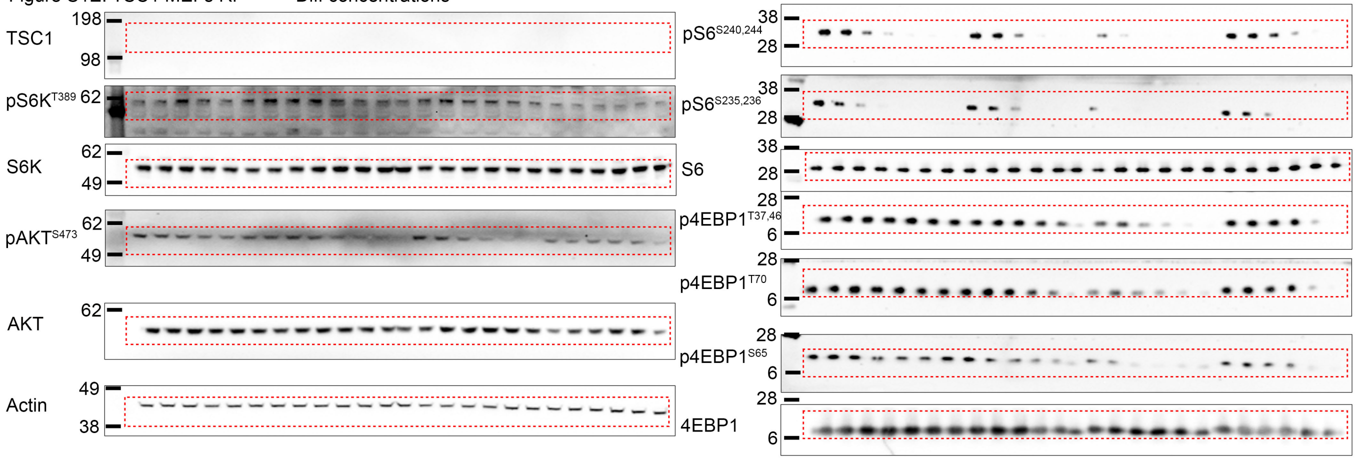

Figure S1F TSC2 MEFs RP Diff Concentrations

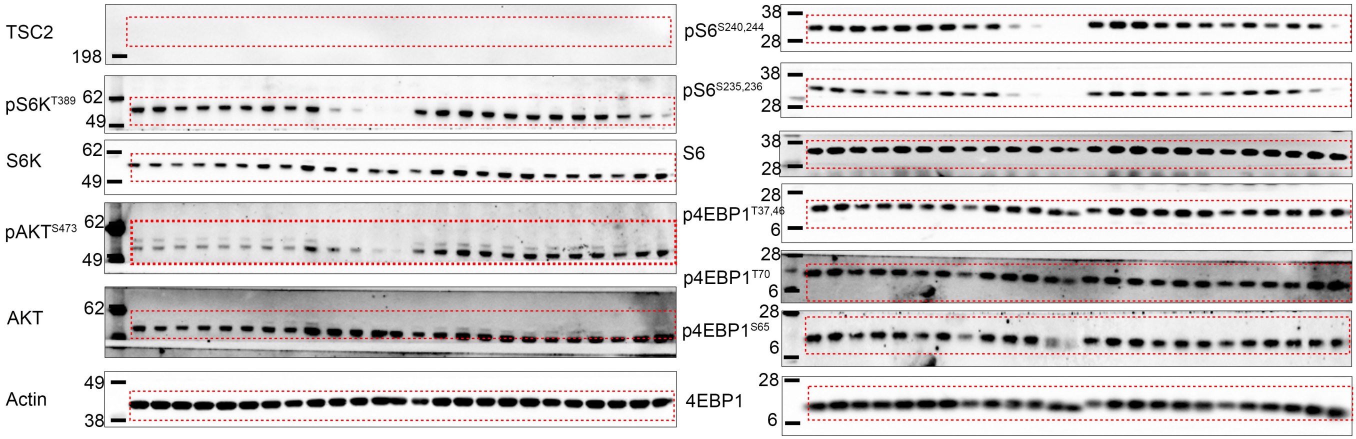

Figure S1G. 705RP Diff concentrations

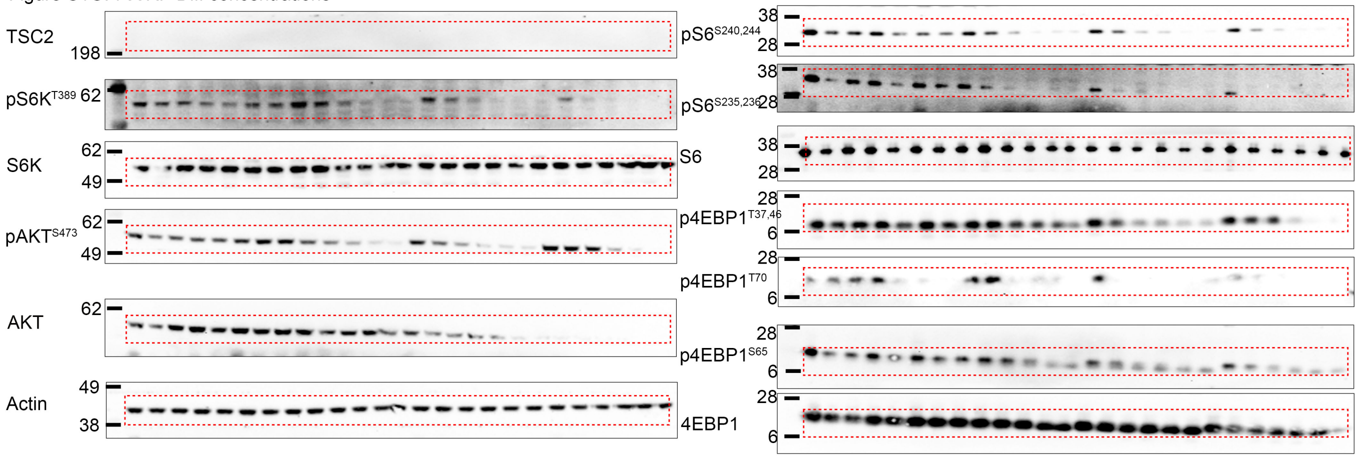

Figure S1H RT4 RP Diff Concentrations

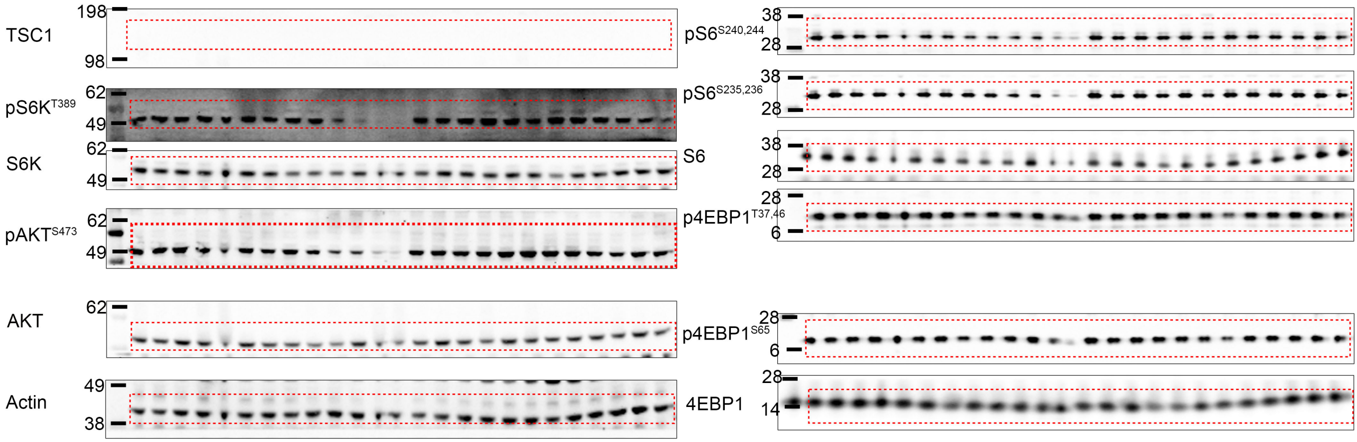

Figure S1I 97-1RP Diff concentrations

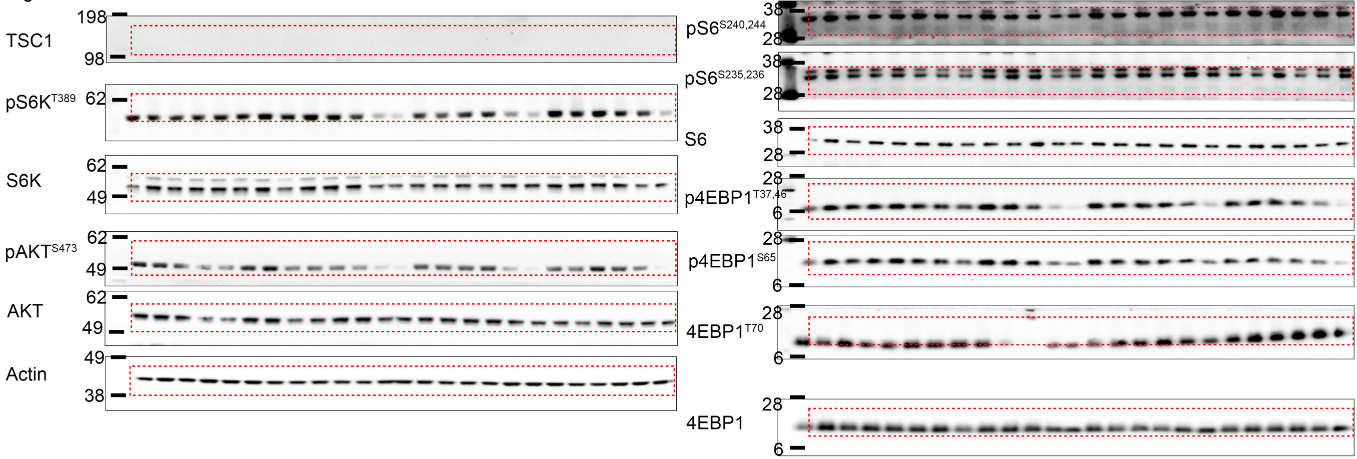

Figure S1J. SNU886 RP Diff concentrations

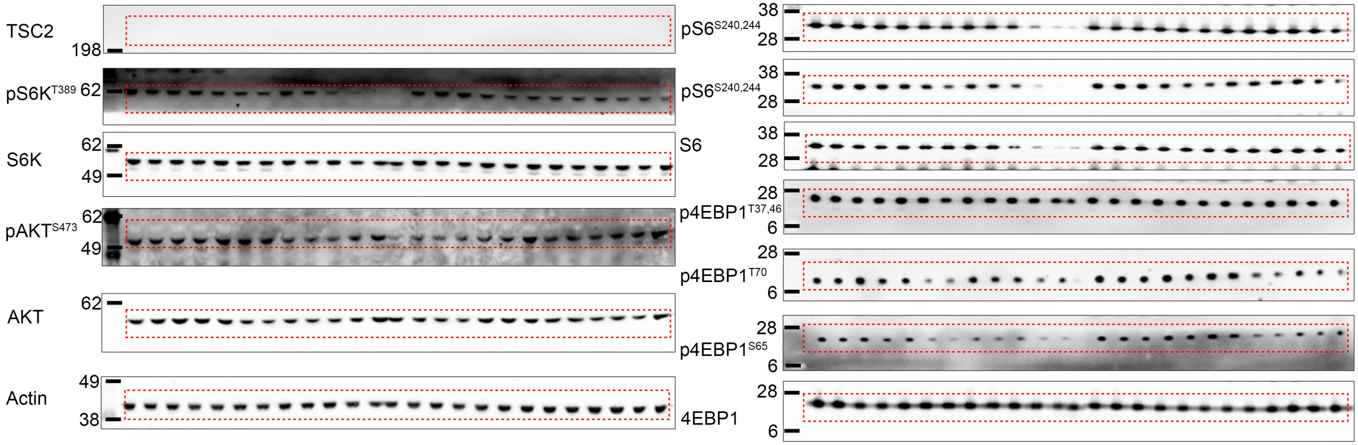

Figure S1K H101RP Diff Concentrations

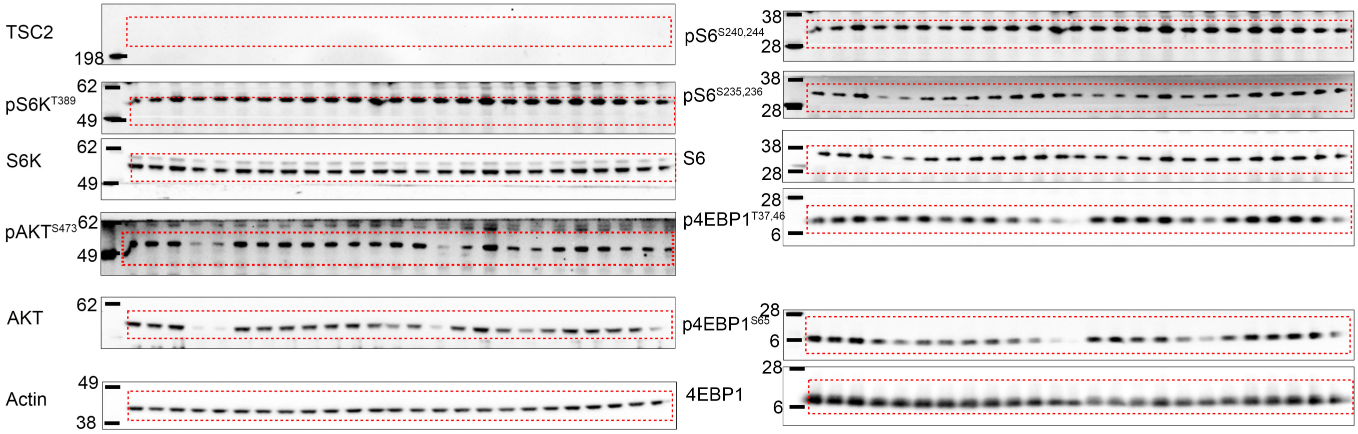

Figure S2E 105KRP Diff concentrations

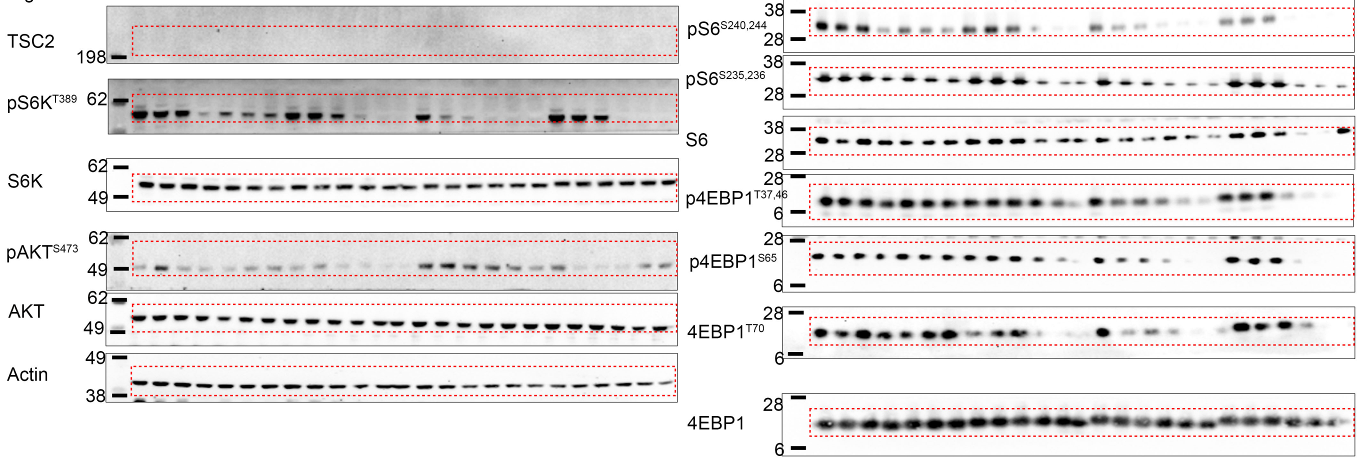

Figure S2A. TSC1 MEFs RP mTOR IP

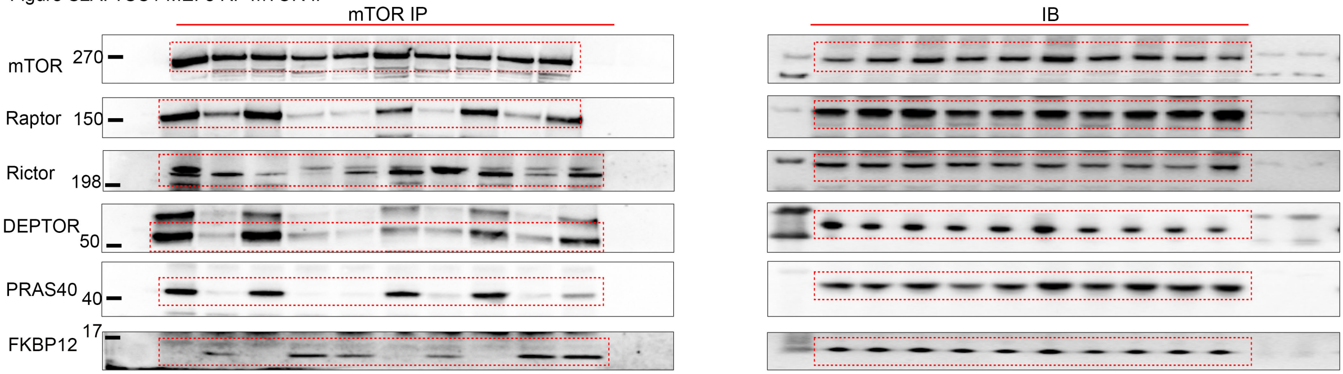

Figure S2B. TSC2 MEFs RP mTOR IP

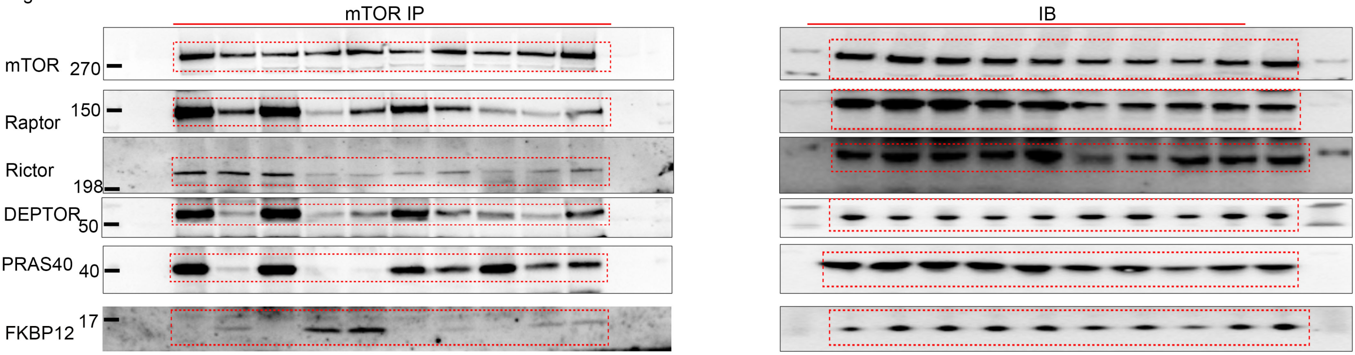

Figure S2C. 705KRP mTOR IP

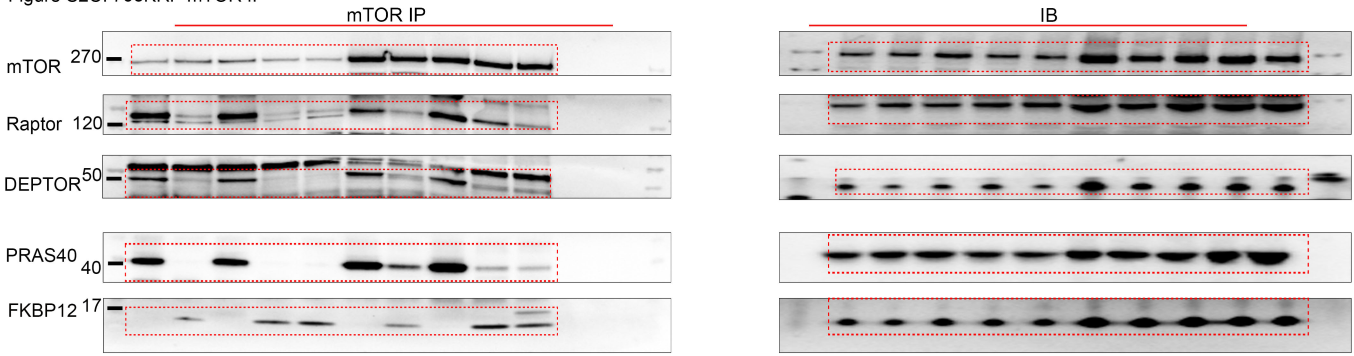

Figure S2D. 97-1RP mTOR IP

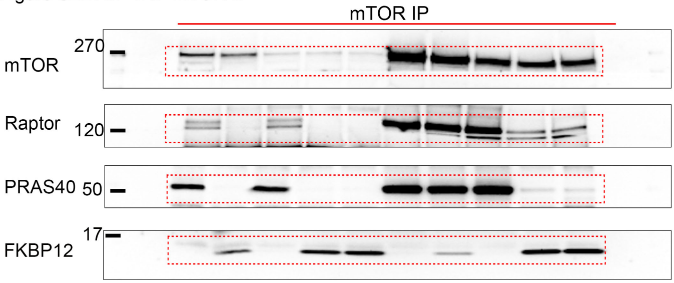

Figure 9A. RP vs Parental cell lines

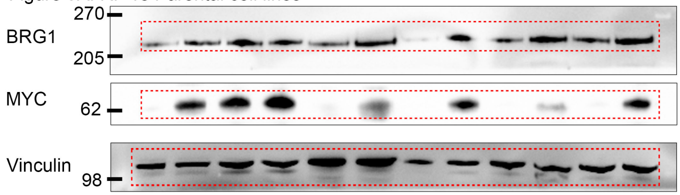

Figure 9B m705RP with siRNA or inhibitors

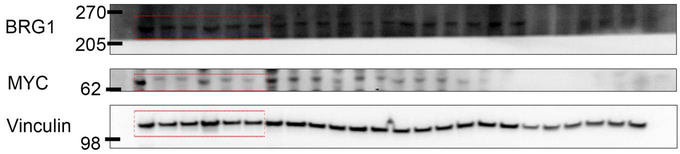

Figure 9D PDX tumors

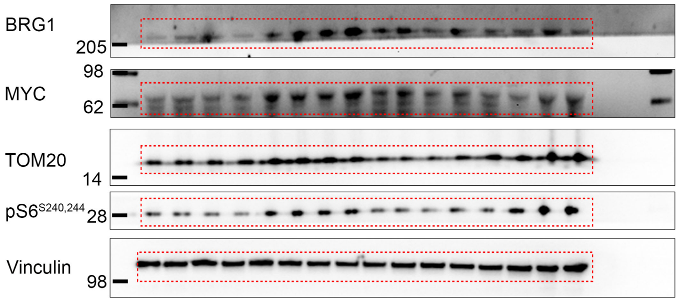

Figure 9C RP treated by inhibitors

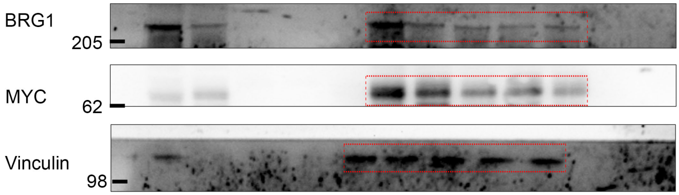

Figure S7A MYC OE

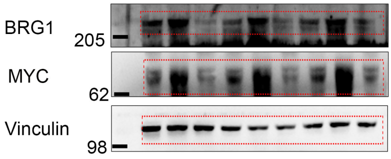

Supplement: Unedited blot and gel images [file jciinsight-10-187448-s023.pdf]
